# Mitochondrial Inner Membrane ABC Transporter Bcmdl1 Is Involved in Conidial Germination, Virulence, and Resistance to Anilinopyrimidine Fungicides in Botrytis cinerea

**DOI:** 10.1128/spectrum.00108-23

**Published:** 2023-06-15

**Authors:** Fei Fan, Yong-Xu Zhu, Min-Yi Wu, Wei-Xiao Yin, Guo-Qing Li, Matthias Hahn, Mohamed S. Hamada, Chao-Xi Luo

**Affiliations:** a National Key Laboratory for Germplasm Innovation and Utilization of Horticultural Crops, Huazhong Agricultural University, Wuhan, China; b College of Plant Science and Technology, Huazhong Agricultural University, Wuhan, China; c Hubei Key Lab of Plant Pathology, College of Plant Science and Technology, Huazhong Agricultural University, Wuhan, China; d Department of Biology, University of Kaiserslautern, Kaiserslautern, Germany; e Pesticides Department, Faculty of Agriculture, Mansoura University, Mansoura, Egypt; University of Debrecen

**Keywords:** *Botrytis cinerea*, anilinopyrimidine, Bcmdl1, mitochondria, ATP synthesis, drug resistance mechanisms

## Abstract

Botrytis cinerea causes gray mold on thousands of plants, leading to huge losses in production. Anilinopyrimidine (AP) fungicides have been applied to control B. cinerea since the 1990s. Although resistance to AP fungicides was detected soon after their application, the mechanism of AP resistance remains to be elucidated. In this study, a sexual cross between resistant and sensitive isolates was performed, and the genomes of parental isolates and progenies were sequenced to identify resistance-related single nucleotide polymorphisms (SNPs). After screening and verification, mutation E407K in the *Bcmdl1* gene was identified and confirmed to confer resistance to AP fungicides in *B. cinerea*. *Bcmdl1* was predicted to encode a mitochondrial protein that belonged to a half-type ATP-binding cassette (ABC) transporter. Although Bcmdl1 was a transporter, it did not mediate resistance to multiple fungicides but mediated resistance specifically to AP fungicides. On the other hand, reductions in conidial germination and virulence were observed in *Bcmdl1* knockout transformants compared to the parental isolate and complemented transformants, illustrating the biological functions of *Bcmdl1*. Subcellular localization analysis indicated that Bcmdl1 was localized in mitochondria. Interestingly, the production of ATP was reduced after cyprodinil treatment in *Bcmdl1* knockout transformants, suggesting that Bcmdl1 was involved in ATP synthesis. Since Mdl1 could interact with ATP synthase in yeast, we hypothesize that Bcmdl1 forms a complex with ATP synthase, which AP fungicides might target, thereby interfering with the metabolism of energy.

**IMPORTANCE** Gray mold, caused by *B. cinerea*, causes huge losses in the production of many fruits and vegetables. AP fungicides have been largely adopted to control this disease since the 1990s, and the development of resistance to AP fungicides initiates new problems for disease control. Due to the unknown mode of action, information on the mechanism of AP resistance is also limited. Recently, mutations in mitochondrial genes were reported to be related to AP resistance. However, the mitochondrial process of these genes remains to be elucidated. In this study, we identified several AP resistance-related mutations by quantitative trait locus sequencing (QTL-seq) and confirmed that mutation E407K in *Bcmdl1* conferred AP resistance. We further characterized the expression patterns, biological functions, subcellular localization, and mitochondrial processes of the *Bcmdl1* gene. This study deepens our understanding of the mechanism of resistance to and mode of action of AP fungicides.

## INTRODUCTION

Botrytis cinerea, the causal agent of gray mold, infects over 1,400 species of plants, including numerous important fruits, vegetables, and ornamentals (1). It can infect leaves, stems, flowers, and fruits preharvest but also causes issues during fruit storage and transport, thus seriously threatening the production and quality of crops. Due to its efficiency, convenience, and economy, chemical control is still the major method used to control this disease. Currently, regularly applied fungicides against gray mold in practice include methyl benzimidazole carbamates (MBCs), dicarboximides (DCFs), quinone outside inhibitors (QoIs), succinate dehydrogenase inhibitors (SDHIs), anilinopyrimidines (APs), and phenylpyrroles (PPs). However, B. cinerea is a classical plant pathogen with a high risk for the development of fungicide resistance and has developed resistance to most of these fungicides all over the world (2–4).

As one major group of botryticides, AP fungicides have been used in agriculture since the 1990s. In total, the three anilinopyrimidines pyrimethanil, cyprodinil, and mepanipyrim are widely commercialized in the crop protection market. However, the mode of action of AP fungicides is still ambiguous (5–7). Previous research found that the inhibition of the mycelial growth of *B. cinerea* was reversed by the addition of methionine (5). Moreover, treatment of mycelia with low concentrations of AP fungicides resulted in an increase in the total amount of free amino acids in *B. cinerea* (8). These results along with the results of radio isotope experiments indicated that AP fungicides might inhibit the biosynthesis of methionine by targeting cystathionine β-lyase (8). However, subsequent research reported that AP fungicides did not inhibit cystathionine β-lyase, and sequence analysis failed to identify resistance-related mutations in the cystathionine β-lyase-encoding gene (6). Furthermore, previous studies focusing on the physiology and biochemistry of AP fungicides demonstrated that they could prevent the secretion of hydrolytic enzymes and cell wall-degrading enzymes but did not contribute much to the identification of the molecular target of AP fungicides (9, 10). Recently, it was observed that all nine identified resistance-conferring proteins were involved in mitochondrial processes, suggesting that AP fungicides primarily target the mitochondria (7).

Although AP fungicides were considered to have a medium risk for the development of resistance (Fungicide Resistance Action Committee [FRAC], https://www.frac.info), resistance to AP fungicides was reported soon after their application. In China, resistance was detected after 3 years of application (11). Based on the resistance level and the sensitivity to fludioxonil, the phenotypes of AP resistance were classified as AniR1, AniR2, and AniR3 (12). AniR1 strains were highly resistant to AP fungicides only. AniR2 and AniR3 strains were weakly resistant to AP fungicides. AniR2 strains were also weakly resistant to the PP fungicide fludioxonil, while AniR3 strains remained sensitive to fludioxonil but displayed low levels of resistance to fenhexamid, a sterol biosynthesis inhibitor (SBI) fungicide. The mechanisms of AniR2 and AniR3 were subsequently identified to be related to the overexpression of the ATP-binding cassette (ABC) transporter gene *BcatrB* and the major facilitator superfamily (MFS) transporter gene *MfsM2*, respectively. Therefore, AniR2 and AniR3 were renamed multidrug resistance 1 (MDR1) and MDR2 (13). Although high-level resistance to AP fungicides was reported soon after their application, the resistance mechanism remained unknown.

Generally, there are four main mechanisms of fungicide resistance (14). First, mutations in the target encoding gene have been demonstrated in many pathogens, causing resistance to many single-site fungicides such as MBCs, DCFs, QoIs, SDHIs, and sterol 14α-demethylase inhibitors (DMIs) (15). Second, the overexpression of the target gene is identified mostly in cases of DMI resistance (16, 17). Third, an enhanced efflux of fungicides is commonly found in *Candida* but also some plant pathogens (18). Finally, the degradation of fungicides is also a mechanism of resistance but has been reported in only a few cases, e.g., the degradation of iprobenfos in the rice blast fungus Pyricularia oryzae and kresoxim-methyl in the apple scab fungus Venturia inaequalis (19, 20). In *B. cinerea*, only two mechanisms of fungicide resistance have been reported so far, including mutation of the target protein and the overexpression of ABC and MFS efflux transporter genes (13, 21).

Due to the unclear mode of action, research on the mechanism of resistance to AP fungicides has been limited. Analysis of ascospore progenies obtained from sexual crosses between resistant and sensitive strains showed that one major gene was probably involved in AniR1 resistance (12). Furthermore, the accumulation of AP fungicides was not reduced in AniR1 strains compared to sensitive ones (12). Based on these results, it was suggested that AP resistance was caused by a mutation in one major gene (*Ani 1*) (6). In a recent study, mutations within nine individual genes were reported to confer AP resistance in *B. cinerea*, among which eight genes, including *Bcmdl1*, were identified from UV laboratory mutants and another gene, *Bcpos5*, was identified from a cross between resistant and sensitive field isolates. Sequencing analysis illustrated that the L412F mutation in *Bcpos5* was the major mutation in resistant field isolates and that the E407K mutation in *Bcmdl1* was the second major mutation (7). The homolog of Bcpos5 is Pos5 in Saccharomyces cerevisiae, an NADH kinase responsible for the detoxification of reactive oxygen species (ROS) (22, 23). The homolog of Bcmdl1 is Mdl1, an ABC transporter in mitochondria exporting peptides produced by the proteolysis of mitochondrial proteins (24). As L412F in *Bcpos5* was the major mutation, Bcpos5 was anticipated to be the primary target of AP fungicides. However, this hypothesis requires further experimentation and direct lines of evidence. Furthermore, little is known about the biological functions and mitochondrial processes of these resistance-related genes in *B. cinerea*.

At present, the development and application of genomics, transcriptomics, and proteomics have facilitated the discovery of unknown genes regulating traits of interest. Quantitative trait locus sequencing (QTL-seq) is a technique that combines bulked-segregant analysis (BSA) and whole-genome resequencing for the rapid identification of interesting quantitative trait loci (QTLs) (25). Using this technique, QTLs of early flowering in cucumbers and 100-seed weight in chickpeas were characterized (26–28). Moreover, association genetics and genome resequencing were also employed to identify avirulence genes in plant pathogens (29, 30). In this study, we used QTL-seq to identify single nucleotide polymorphisms (SNPs) responsible for AP resistance, and the E407K mutation in *Bcmdl1* was successfully identified, consistent with the results of a recent study (7). Focusing on this gene, experiments were conducted, and the results enhanced our understanding of the mechanism of resistance to as well as the mode of action of AP fungicides.

## RESULTS

### Seven SNPs were identified to be related to AP resistance by QTL-seq.

**FIG 1:**
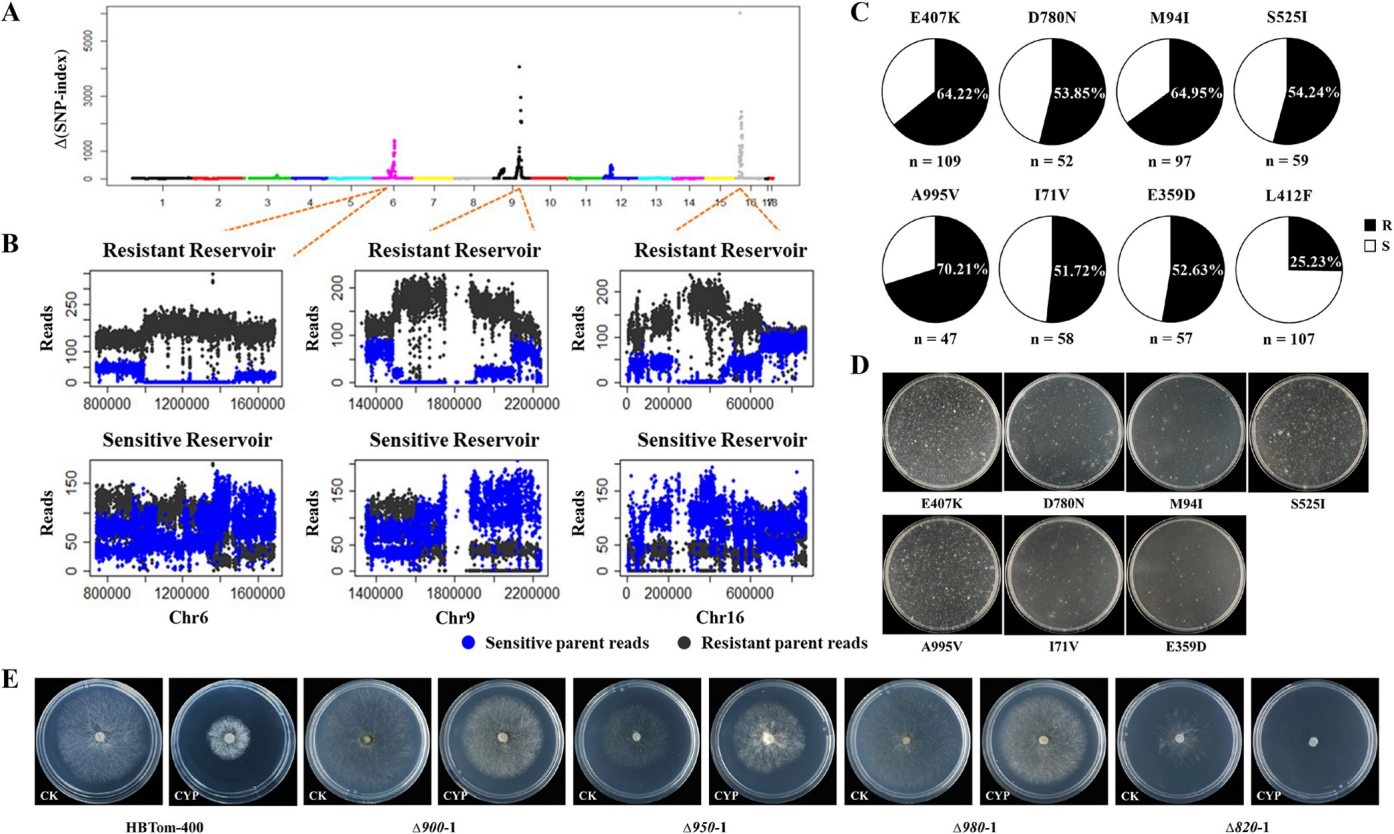
Mutation E407K in *Bcmdl1* was identified as being related to anilinopyrimidine resistance. (A) Plots of Δ(SNP index) values in the whole genome. The whole genome was analyzed using 50 kb as a window and 5 kb as a step. The Δ(SNP-index) value was calculated by the subtraction of the SNP index values between resistant and sensitive bulk pools. (B) Plots of resistant and sensitive reads in the resistant and sensitive reservoirs in the three candidate QTL regions, respectively. (C) Frequency of candidate resistance-related SNPs as inferred from Sanger sequencing within anilinopyrimidine-resistant isolates collected from strawberries and tomatoes in 2012 and 2013. E407K and D780N were mutations in *Bcin16g00820* (*Bcmdl1*). M94I was a mutation in *Bcin16g00900*. S525I and A995V were mutations in *Bcin16g00950*. I71V and E359D were mutations in *Bcin16g00980*. L412F was a mutation in *Bcpos5*, a known mutation responsible for anilinopyrimidine resistance. R, resistant; S, sensitive. (D) Confirmation of candidate resistance-related SNPs by homologous recombination. Candidate mutations along with both flanking regions of ~500 bp were transformed into sensitive isolates using PEG-mediated protoplast transformation, and cyprodinil was used as the selective agent. The colonies were observed after ~12 days of transformation at 20°C in the dark. (E) Sensitivity of knockout transformants to cyprodinil. Isolate HBTom-400 is the parental isolate, and transformants Δ*900-1*, Δ*950-1*, Δ*980-1*, and Δ*820-1* are the knockout transformants of *Bcin16g00900*, *Bcin16g00950*, *Bcin16g00980*, and *Bcin16g00820* (*Bcmdl1*), respectively. CK and CYP are fresh Czapek-Dox agar and Czapek-Dox agar amended with cyprodinil at 10 μg/mL, respectively. The colonies were observed after 3 days of incubation at 20°C in the dark.

To identify AP resistance-related SNPs, QTL-seq was used in this study. A sexual cross performed between the cyprodinil-resistant isolate HBTom-400 and the cyprodinil-sensitive isolate HBTom-103 produced 23 progenies, and their resistance to cyprodinil was tested on Czapek-Dox agar (CzA) plates with a discriminatory dose of 10 μg/mL (7). The results showed that 6 of the progenies were sensitive to cyprodinil, while the other 17 were resistant. The genomic DNAs of all six sensitive progenies and seven randomly selected resistant progenies were extracted and mixed at an equal ratio. The bulk DNAs and the DNAs of the parental isolates were then resequenced using an Illumina genome analyzer. Finally, BSA was performed to process the genome sequencing data. As a result, resistance-related SNPs were identified primarily at chromosomes 6, 9, and 16 (Fig. 1A and B).

Theoretically, resistance-related SNPs should be nonsynonymous mutations and should exist only in the resistant parental isolate and the resistant progeny pool. According to these rules, 29 SNPs in 11 genes were identified as potential resistance-related SNPs (see Table S1 in the supplemental material). The potential SNPs were further investigated in resistant and sensitive field isolates by Sanger sequencing to eliminate false positives. In total, 109 resistant isolates and 35 sensitive isolates were sequenced. Eventually, seven SNPs in four genes were found to be related to AP resistance, including mutations E407K and D780N in *Bcin16g00820* (*Bcmdl1*), mutation M94I in *Bcin16g00900*, mutations S525I and A995V in *Bcin16g00950*, and mutations I71V and E359D in *Bcin16g00980*. Moreover, the frequency of each SNP was >50% (Fig. 1C and Table S2), indicating that these seven SNPs were highly relevant to AP resistance. Mutation L412F in *Bcpos5* is the major known mutation conferring AP resistance in Europe (7), and its frequency in this study was only ~25% (Fig. 1C and Table S2).

### Mutation E407K in *Bcmdl1* conferred resistance to AP fungicides.

The relationships between the candidate resistance-related SNPs and AP resistance were further validated by genetic transformation. The candidate SNPs, including flanking regions of ~500 bp upstream and downstream, were amplified from resistant isolate HBTom-400, and each fragment was transformed into sensitive isolate HBTom-103 using polyethylene glycol (PEG)-mediated protoplast transformation. After the transformation of PCR fragments containing E407K in *Bcmdl1* or S525I or A995V in *Bcin16g00950*, many colonies appeared under selection with cyprodinil, while only a few colonies were observed for the rest of the candidate mutations (Fig. 1D). After three rounds of selection with cyprodinil at 20 μg/mL, only a few transformants could survive, and most colonies were obtained from fragments with the E407K, S525I, and A995V mutations (Table S3). In addition, the potential SNPs were further validated by Sanger sequencing, and only mutation E407K was confirmed in the transformants by sequencing (Table S3), indicating the necessity of sequencing after cyprodinil screening. Furthermore, the *Bcin16g00900*, *Bcin16g00950*, *Bcin16g00980*, and *Bcin16g00820* (*Bcmdl1*) genes were each knocked out in the resistant isolate HBTom-400. As expected, all of the transformants except those from the knockout of *Bcmdl1* were as resistant as the parental isolate (Fig. 1E). These results of PCR fragment transformation demonstrated that only E407K in *Bcmdl1* was involved in resistance to AP fungicides.

**FIG 2:**
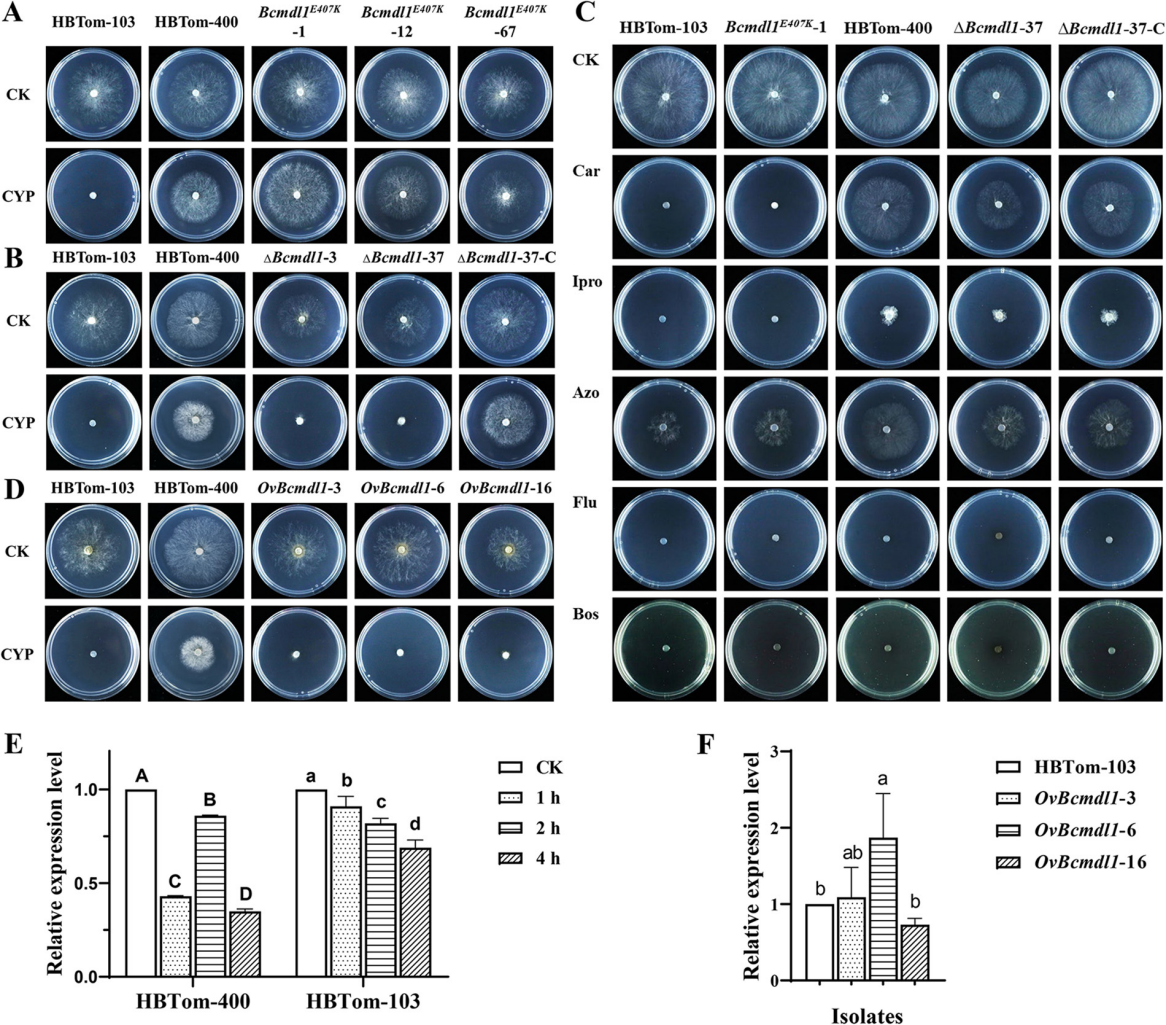
Mutation E407K in *Bcmdl1*, rather than overexpression, specifically confers resistance to anilinopyrimidine fungicides. (A) Sensitivity of E407K insertion transformants to cyprodinil. Isolates HBTom-400 and HBTom-103 are field isolates that are resistant and sensitive to anilinopyrimidine fungicides, respectively. Transformants *Bcmdl1^E407K^*-1, -12, and -67 are E407K insertion transformants obtained by PEG-mediated protoplast transformation using the whole gene as the DNA construct. CK and CYP are fresh Czapek-Dox agar and Czapek-Dox agar amended with cyprodinil at 10 μg/mL, respectively. The colonies were observed after 3 days of incubation at 20°C in the dark. (B) Sensitivity of *Bcmdl1* knockout transformants to cyprodinil. Transformants Δ*Bcmdl1*-3 and -37 are knockout transformants of *Bcmdl1* with HBTom-400 as the parental isolate. Transformant Δ*Bcmdl1*-37C is the complemented transformant of *Bcmdl1* using transformant Δ*Bcmdl1*-37 as the parental strain. (C) Sensitivities of E407K insertion and *Bcmdl1* knockout transformants to different fungicides. CK is fresh potato dextrose agar. Car, Ipro, Azo, and Flu are potato dextrose agar amended with the corresponding fungicides at 1 μg/mL carbendazim, 5 μg/mL iprodione, 10 μg/mL azoxystrobin, and 0.4 μg/mL fludioxonil, respectively, and Bos is yeast Bacto acetate agar amended with boscalid at 75 μg/mL. (D) Sensitivity of *Bcmdl1* overexpression transformants to cyprodinil. Transformants *OvBcmdl1*-3, -6, and -16 are overexpression transformants of *Bcmdl1* with HBTom-103 as the parental isolate by PEG-mediated protoplast transformation using the promoter region of *EF-1* as the DNA construct. (E) Relative transcription levels of *Bcmdl1* in HBTom-400 and HBTom-103 after cyprodinil treatment. The relative expression levels of *Bcmdl1* in each isolate after cyprodinil treatment for 1, 2, and 4 h are the relative expression levels in the corresponding isolates without cyprodinil treatment (CK) determined using RT-qPCR. The β-tubulin gene was selected as the reference gene. Different letters indicate significant differences (*P* < 0.05) according to one-way analysis of variance (ANOVA) using the least significant difference (LSD) test. Means and standard errors were calculated from three repeats. (F) Relative expression levels of *Bcmdl1* in *Bcmdl1* overexpression transformants. The relative expression level of *Bcmdl1* in each transformant is the relative expression level compared with that of the parental isolate. Means and standard errors were calculated from three repeats. Statistical analyses were performed using a paired *t* test (*P* < 0.05).

To further confirm the relationship between E407K in *Bcmdl1* and AP resistance, the whole sequence of *Bcmdl1* from resistant isolate HBTom-400 was transformed into sensitive isolate HBTom-103 using PEG-mediated protoplast transformation (Fig. S1). During the transformation process, hygromycin B selective medium was used, which avoided unrelated mutations of AP resistance. As a result, three homozygous transformants grew as well as the resistant isolate HBTom-400 on CzA plates amended with 10 μg/mL cyprodinil, while the parental isolate HBTom-103 could not grow on these plates (Fig. 2A). The sensitivity assay also showed that the median effective concentration (EC_50_) values for the three homozygotes were ~20 μg/mL, which were similar to that of resistant isolate HBTom-400 but about 100 times higher than that of parental isolate HBTom-103 (Table S4). Taken together, these results indicated that mutation E407K in *Bcmdl1* conferred resistance to AP fungicides in *B. cinerea*. On the other hand, the *Bcmdl1* gene was knocked out in resistant isolate HBTom-400 (Fig. S2) and complemented (Fig. S3). As expected, the knockout transformants could not grow on CzA plates containing 10 μg/mL cyprodinil, just like the sensitive isolate HBTom-103, while the parental isolate HBTom-400 and the complemented transformant Δ*Bcmdl1-*37C grew normally, indicating a loss of resistance in the knockout transformants (Fig. 2B). The EC_50_ values for the three knockout transformants were ~0.02 μg/mL, which were similar to that of sensitive isolate HBTom-103 and about 100 times lower than that of parental isolate HBTom-400 (Table S4). Besides cyprodinil, the sensitivity of *Bcmdl1*-related transformants to pyrimethanil was also tested, and results similar to those for sensitivity to cyprodinil were observed (Fig. S4). In summary, the *Bcmdl1* gene containing the E407K mutation conferred resistance to AP fungicides in *B. cinerea*.

In addition to AP fungicides, the resistance of different strains to carbendazim (MBC), iprodione (DCF), azoxystrobin (QoI), boscalid (SDHI), and fludioxonil (PP) was also tested. Similar to the parental isolate HBTom-103, the transformants with the E407K mutation maintained sensitivity profiles similar to those for other fungicides tested (Fig. 2C). Moreover, the knockout transformants still showed the same sensitivity to other fungicides as that of the parental isolate HBTom-400 and the complemented transformant Δ*Bcmdl1-*37C (Fig. 2C). These results demonstrated that the insertion of the E407K mutation and the knockout of *Bcmdl1* had no influence on resistance to other classes of fungicides except for AP fungicides, indicating that *Bcmdl1* specifically contributed to AP resistance.

Since Bcmdl1 was predicted to be an ABC transporter, the expression pattern of *Bcmdl1* in both sensitive and resistant isolates after treatment with cyprodinil for 1, 2, and 4 h was determined by reverse transcription-quantitative PCR (RT-qPCR). The results showed that no upregulation but downregulation of *Bcmdl1* was observed in either the resistant or sensitive isolates after cyprodinil treatment (Fig. 2E). Additionally, we attempted to overexpress *Bcmdl1* by fusing the promoter region of the *EF-1* gene with the coding region of *Bcmdl1*, and this DNA construct was transformed into sensitive isolate HBTom-103 (Fig. S5). Even though the expression level of *Bcmdl1* in the *OvBcmdl1*-6 transformant was significantly increased (Fig. 2F), this transformant remained as sensitive to cyprodinil as the parental isolate (Fig. 2D). These results illustrated that resistance to AP fungicides is not related to the overexpression of *Bcmdl1* in *B. cinerea*.

### Bcmdl1 is a typical and conserved ABC transporter.

**FIG 3:**
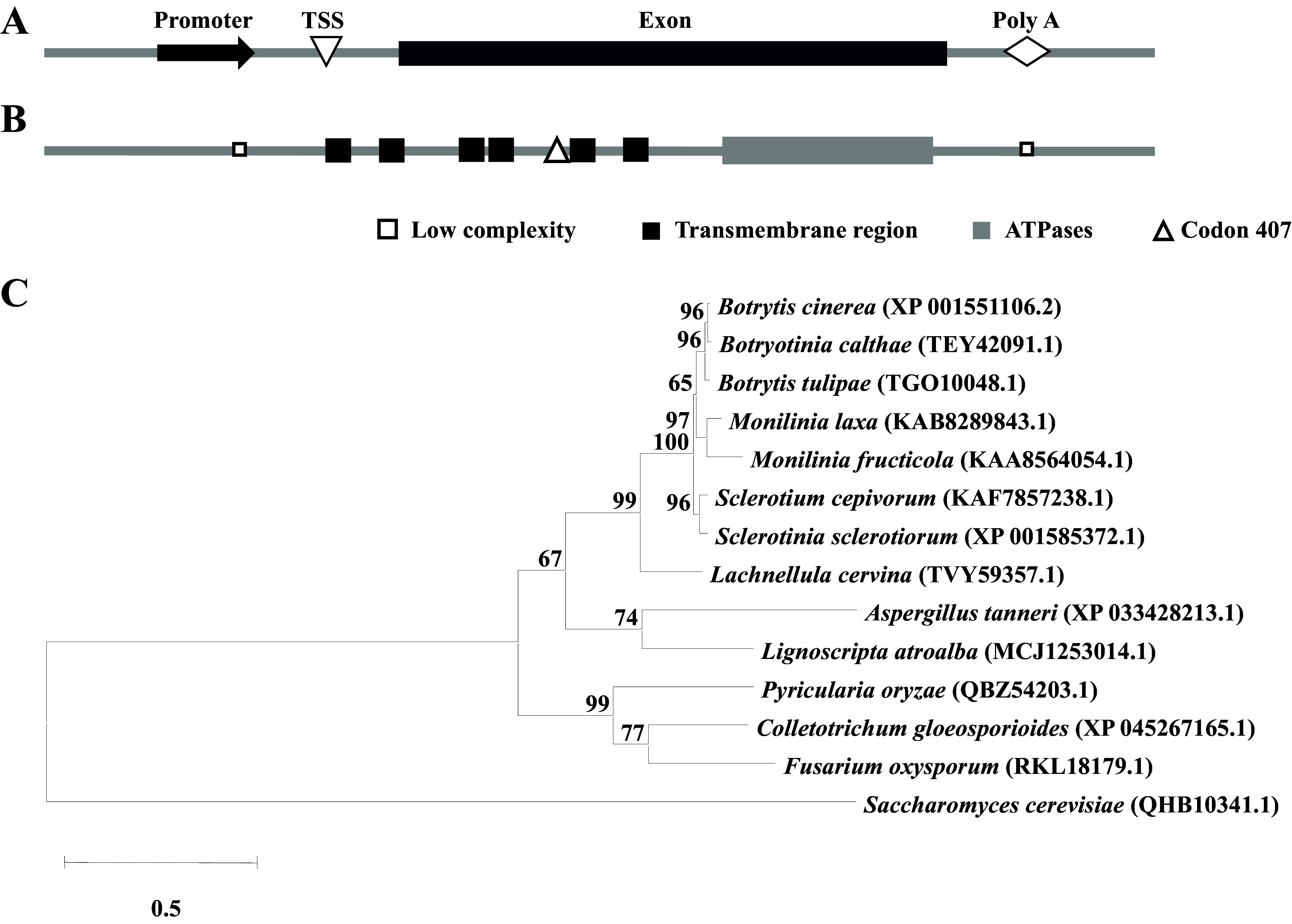
Bcmdl1 is a classical and conserved ABC transporter. (A) Structure of the *Mdl1* gene of Botrytis cinerea (*Bcmdl1*). TSS, transcriptional start site; Poly A, poly(A) tail. The figure is not drawn to scale. (B) Domain analysis of Bcmdl. The analysis was performed using SMART (http://smart.embl-heidelberg.de/). The figure is not drawn to scale. (C) Phylogenetic analysis of Mdl1 homologs from different fungal species. The phylogenetic tree was constructed with MEGA 7 software using the maximum likelihood method.

Bioinformatic analysis was performed to predict the function of Bcmdl1. Alignment of the genomic DNA (gDNA) and cDNA sequences of the *Bcmdl1* gene revealed that it contains a 2,418-bp (including the stop codon) coding sequence without an intron, encoding a protein composed of 805 amino acids (Fig. 3A). Domain analysis predicted that Bcmdl1 was composed of six transmembrane regions and an ATPase domain at the C terminus (Fig. 3B), which was the classical component of a half-type ABC transporter. Notably, the resistance mutation E407K occurred next to a transmembrane region. Phylogenetic analysis of homologous proteins of Bcmdl1 revealed that Mdl1 proteins are widely distributed in ascomycetes and highly conserved in Sordariomycetes, including Aspergillus, *Pyricularia*, *Colletotrichum*, and Fusarium; e.g., the amino acid sequence of Bcmdl1 showed 97.91% and 62.39% identities to the homologs in Botryotinia calthae (GenBank accession number TEY42091.1) and Fusarium oxysporum (accession number RKL18179.1) (Fig. 3C and Fig. S6).

### *Bcmdl1* is involved in conidial germination and virulence.

**FIG 4:**
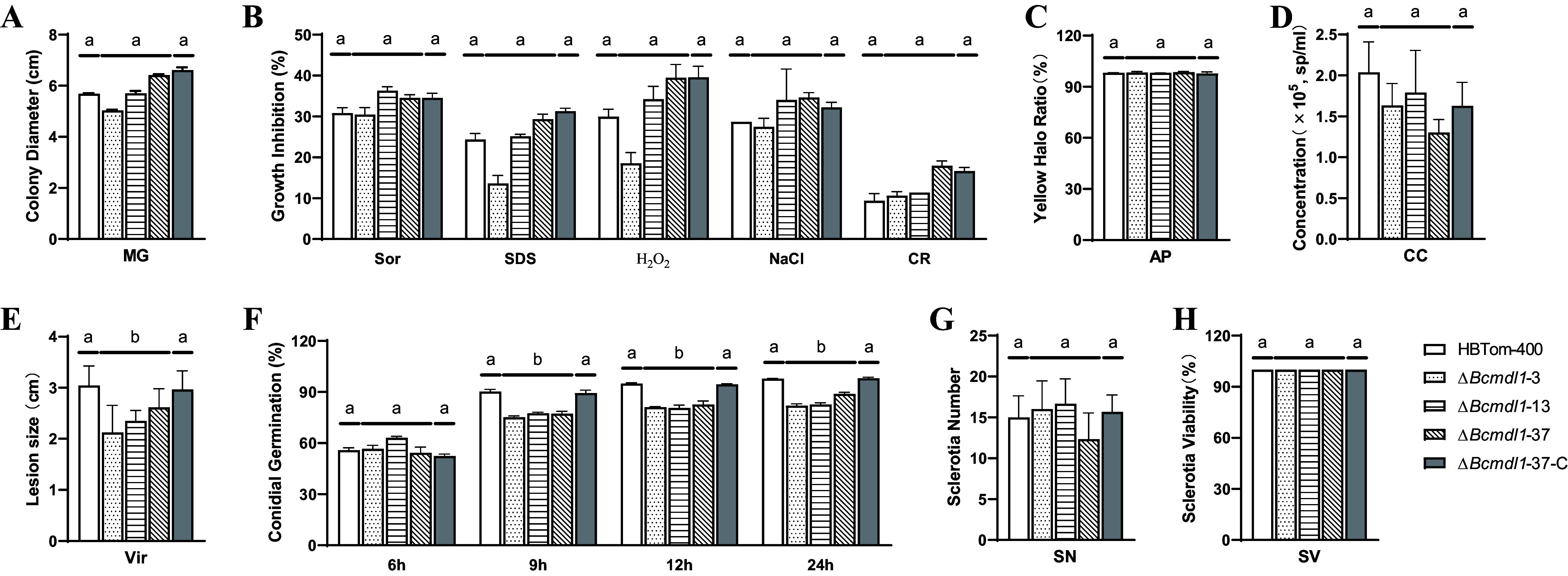
Bcmdl1 is involved in conidial germination and virulence. (A) Mycelial growth of *Bcmdl1* knockout transformants. Mycelial growth was measured after incubation on potato dextrose agar for 3 days at 20°C in the dark. (B) Sensitivities of *Bcmdl1* knockout transformants to different chemicals. Sensitivities to sorbitol (Sor), sodium dodecyl sulfate (SDS), H_2_O_2_, NaCl, and Congo red (CR) were determined on potato dextrose agar amended with the corresponding chemicals at 1.2 M, 0.01% (wt/vol), 5 mM, 0.5 M, and 300 μg/mL, respectively. The colony diameter was determined after 3 days of incubation at 20°C in the dark. Sensitivity is expressed as mycelial growth inhibition. (C) Acid production (AP) by *Bcmdl1* knockout transformants. Acid production was examined on potato dextrose agar containing 0.005% (wt/vol) bromophenol blue after incubation for 3 days at 20°C in the dark and is expressed as the ratio of the diameter of the colony to the diameter of the yellow halo. (D) Sporulation of *Bcmdl1* knockout transformants. Sporulation was estimated on potato dextrose agar after 1 month of incubation at 20°C in the dark. CC, conidial concentration; Sp, spores. (E) Virulence (Vir) of *Bcmdl1* knockout transformants. Tomato fruit inoculated with a conidial suspension was used to evaluate virulence. Virulence was indicated by the lesion size after incubation at room temperature for 5 days. (F) Conidial germination (CG) of *Bcmdl1* knockout transformants. Conidial germination was analyzed on 1.5% water agar after incubation for 6, 9, 12, and 24 h. (G) Sclerotial production by *Bcmdl1* knockout transformants. Sclerotial production was measured on potato dextrose agar after 1 month of incubation at 20°C in the dark. SN, sclerotial number. (H) Sclerotial viability (SV) of *Bcmdl1* knockout transformants. Sclerotial viability was determined on potato dextrose agar after storage at 4°C for 3 months. HBTom-400 was the parental isolate. The transformants Δ*Bcmdl1*-3, -13, and -37 are knockout transformants of *Bcmdl1* with HBTom-400 as the parental isolate. Transformant Δ*Bcmdl1*-37C is the complemented transformant of *Bcmdl1* using transformant Δ*Bcmdl1*-37 as the parental strain. Different letters indicate significant differences (*P* < 0.05) according to one-way analysis of variance (ANOVA) using the least significant difference (LSD) test. Means and standard errors were calculated from three repeats. Percentage data and sporulation data were arcsine and logarithm transformed before analysis, respectively.

*Bcmdl1* is unlikely to be the primary target of AP fungicides because some resistant isolates do not contain the E407K mutation in *Bcmdl1* (Fig. 1C and Table S2) but rather a mediator between the primary target and resistance to AP fungicides. For further investigation of the function of *Bcmdl1*, phenotypic characterization of the knockout transformants was performed (Fig. 4). Compared to the parental and complemented strains, the rate of germination of conidia in *Bcmdl1* knockout transformants was significantly reduced on water agar after incubation for 9, 12, and 24 h, while the germination rate after 6 h was similar (Fig. 4F) (*P* < 0.05). At the same time, the lesion size caused by knockout transformants was significantly smaller than those of the parental isolate and the complemented strains (Fig. 4D) (*P* < 0.05). There was no significant difference among the parental and complemented strains and the knockout transformants in mycelial growth, sporulation, sclerotial production and viability, acid production, and sensitivity to NaCl, sorbitol, sodium dodecyl sulfate (SDS), hydrogen peroxide (H_2_O_2_), and Congo red (CR) (*P* > 0.05) (Fig. 4). These results showed that *Bcmdl1* was related to conidial germination and virulence in *B. cinerea*.

### Bcmdl1 is localized in the mitochondrial membrane and is involved in ATP synthesis.

**FIG 5:**
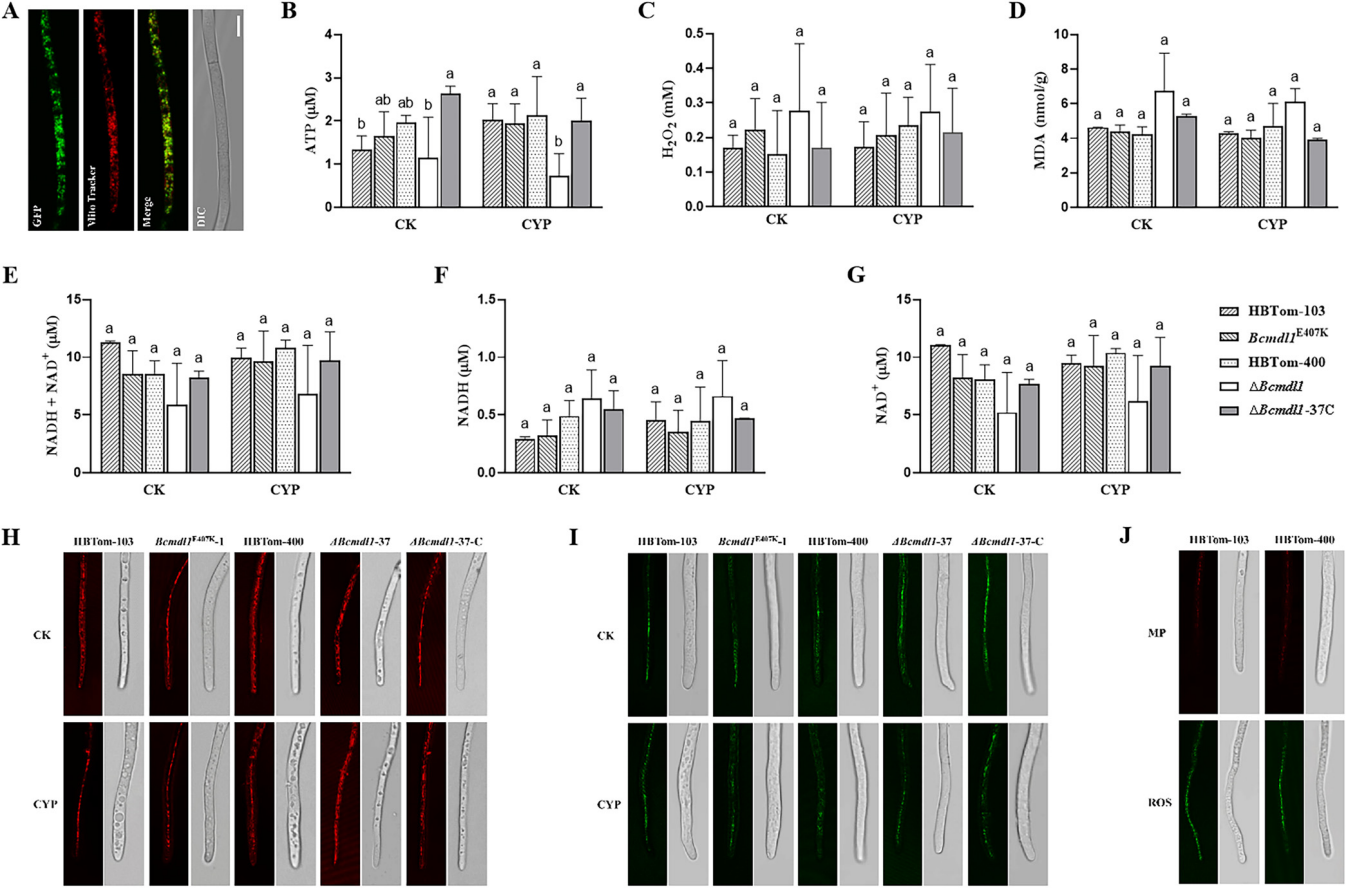
The mitochondrial ABC transporter Bcmdl1 contributes to ATP synthesis. (A) Colocalization of Bcmdl1 with the mitochondrial dye Mito-Tracker. Bcmdl1::GFP was ectopically expressed in isolate HBTom-103 and then examined using a Leica TCS SP8 confocal microscope. DIC, differential interference contrast. (B to I) Contents of ATP (B), hydrogen peroxide (H_2_O_2_) (C), malondialdehyde (MDA) (D), NAD^+^/NADH (E), the NAD reduced form (NADH) (F), the NAD oxidized form (NAD^+^) (G), mitochondrial membrane potential (MP) (H), and reactive oxygen species (ROS) (I) in knockout and E407K insertion transformants of *Bcmdl1* with or without cyprodinil treatment. (J) Positive controls for ROS and MP using parental isolates. The cell-permeant reagent 2′,7′-dichlorofluorescein diacetate (DCFH-DA) was used as the positive control for ROS at 10 mM for 2 h. Carbonyl cyanide *m*-chlorophenylhydrazone (CCCP) was used as the positive control for MP at 10 mM for 4 h. HBTom-103 and HBTom-400 were the parental isolates of the E407K insertion and knockout transformants of *Bcmdl1*, respectively. The *Bcmdl1^E407K^* group included three E407K insertion transformants, *Bcmdl1^E407K^*-1, -12, and -67. The Δ*Bcmdl1* group included three knockout transformants, Δ*Bcmdl1*-3, -13, and -37. Transformant Δ*Bcmdl1*-37C is the complemented transformant of *Bcmdl1*. CK is the control without cyprodinil treatment, whereas CYP is treatment with cyprodinil. Different letters indicate significant differences (*P* < 0.05) according to one-way analysis of variance (ANOVA) using the least significant difference (LSD) test. Means and standard errors were calculated from three repeats.

To confirm the localization of Bcmdl1, a Bcmdl1::green fluorescent protein (GFP) fusion protein was transformed into *B. cinerea* (Fig. S7). The fluorescence was localized in ovoid bodies distributed in the cytoplasm, which was coincident with staining with the mitochondrion-specific dye Mito-Tracker red Chloromethyl-X-rosamine (CMXRos), suggesting that Bcmdl1 was also localized in mitochondria (Fig. 5A). To investigate the mitochondrion-associated functions of Bcmdl1, several parameters of mitochondria were compared between wild-type (WT) isolates and *Bcmdl1* mutants, including the contents of ATP, NAD^+^/NADH, reactive oxygen species (ROS), malondialdehyde (MDA), and H_2_O_2_. Under normal conditions, the contents of ATP were not significantly different among the *Bcmdl1* knockout transformants, the E407K insertion transformants, and the WT isolate. However, ATP production was significantly inhibited in the knockout transformants compared to the WT parental isolate and the complemented transformant in the presence of cyprodinil (Fig. 5B). This result indicated that Bcmdl1 was involved in ATP synthesis, and AP fungicides could emphasize the role of Bcmdl1 in ATP synthesis. Nevertheless, the contents of H_2_O_2_ (Fig. 5C), MDA (Fig. 5D), NAD^+^/NADH (Fig. 5E to G), and ROS (Fig. 5I and Fig. S8A) were not significantly altered in both the *Bcmdl1* knockout and E407K insertion transformants compared to the corresponding parental isolates, indicating that Bcmdl1 was not directly related to the tricarboxylic acid cycle, lipid peroxidation, and oxidative stress (OS). To further explore how Bcmdl1 influences ATP synthesis, the mitochondrial membrane potential (MP) was determined using the fluorescence probe JC-1, which shows red fluorescence for high MP. The results showed that there was no significant difference between the various transformants and their corresponding parental isolates (Fig. 5H and Fig. S8B), indicating that Bcmdl1 is not involved in electron transfer. Taken together, these results suggested that the mitochondrial ABC transporter Bcmdl1 might be involved in ATP synthesis by influencing ATP synthase activity and that AP fungicides might target the metabolism of ATP.

## DISCUSSION

As a relatively new class of fungicides with a novel mode of action, pyrimethanil, cyprodinil, and other anilinopyrimidines have been applied in crop protection since the 1990s. At present, AP fungicides are considered potential inhibitors of methionine biosynthesis (FRAC). However, the real mode of action of AP fungicides remains to be reclassified (5). Due to their broad spectrum and excellent activity, AP fungicides were used against many ascomycetes and basidiomycetes, including *B. cinerea* (31). Although the risk of resistance development was considered medium, resistance to AP fungicides in *B. cinerea* was recorded worldwide soon after their application (2, 3, 11, 32). As a result of the unknown mode of action, progress in the determination of the mechanism of resistance to AP fungicides was limited. In general, AP resistance was possibly controlled by a mutation in one major gene of mitochondria (6, 7, 12).

In our previous studies, AP resistance was widely detected in *B. cinerea* isolated from greenhouse strawberries and tomatoes in Hubei Province, China (4, 33). In order to make effective antiresistance strategies, it would be beneficial to know the mechanism of resistance to AP fungicides. Since many avirulence genes were identified successfully by genetic mapping (34, 35), in this study, a sexual cross between AP-resistant and -sensitive isolates was conducted to identify the gene conferring resistance to AP fungicides. As a result, altogether, 23 progenies were obtained, including 6 sensitive isolates and 17 resistant isolates. In previous studies, the ratio of resistant to sensitive progenies was nearly 1:1 in 142 progenies (72:70) (7, 36). The 1:1 ratio was also supported by another study (12). Unlike the hundreds of progenies obtained in previous studies, only 23 progenies were isolated in this study, which was not suitable to calculate the ratio of resistant to sensitive progenies. Nevertheless, such a cross did not affect our study because we used these progenies only to make sequencing pools to identify resistance-related SNPs rather than to map certain traits.

Evidence of genetic transformation is important to validate AP resistance-related SNPs discovered by QTL-seq. There are two kinds of transformation methods according to the DNA constructs used. One method is to use partial gene sequences containing candidate SNPs, and the other is to use the whole sequence. For transformation with partial sequences, the AP fungicide cyprodinil was used as the selection agent, which might cause false positives because transformants could generate mutations of irrelevance under the selection pressure of an AP fungicide during the process. Therefore, other rounds of AP selection and Sanger sequencing are needed to reduce the number of false positives. In contrast, this problem does not exist using transformation with the whole sequence where hygromycin is used as the selection agent, providing a better way to confirm the relationship between candidate SNPs and AP resistance. In a previous study, resistance-related SNPs were confirmed by transformation using partial gene sequences (7). In this study, the relationship between candidate SNPs and AP resistance was first validated by the transformation of gene fragments. Later, the positive SNP (only E407K in *Bcmdl1*) was further verified by the transformation of the complete gene sequence. The reason why only the *Bcmdl1* gene was verified by genetic transformation with the whole sequence is that other methods, e.g., partial sequence transformation, had already shown negative results. Above all, except for one candidate SNP in *Bcin16g00900*, there were two candidate SNPs in each of the *Bcmdl1*, *Bcin16g00950*, and *Bcin16g00980* genes, and it was dramatically difficult to obtain the whole gene sequence containing the single candidate SNP. We tried fusion and site-directed mutagenesis, but the results were not satisfying. Therefore, we did not verify each candidate SNP using the whole gene sequence including mutation E407K in *Bcmdl1*.

The *Bcmdl1* gene is the homolog of *Mdl1* in yeast. The *Mdl1* gene showed considerable similarity to the mammalian P-glycoprotein/multidrug resistance and peptide transporter genes, and it was not essential for the viability of yeast (37). Even though Mdl1 was named multidrug resistance-like protein, few reports were found regarding its function in multidrug resistance. Instead, Mdl1 was a half-type ABC transporter in the mitochondrial inner membrane, exporting peptides generated upon the proteolysis of mitochondrial proteins by the m-AAA (matrix-ATPases associated with a variety of cellular activities) protease (24). However, perhaps due to the specificity of the peptides that Mdl1 recognized, a subsequent study failed to confirm the peptide transport activity or peptide-stimulated ATPase activity of Mdl1 (38). In addition, Mdl1 might share a substrate with Atm1, another ABC transporter in the inner membrane of mitochondria, because Mdl1 could complement Atm1 function (39). Mdl1 also played an important role in the regulation of cellular resistance to oxidative stress because the overexpression of Mdl1 led to increased resistance to oxidative stress (40). Above all, Mdl1 usually forms a homodimeric complex. At low ATP levels, it formed a complex with F_1_F_o_-ATP synthase, but at high ATP levels, it dissociated from the synthase, indicating that Mdl1 was involved in cellular energy metabolism (41).

Similar to Mdl1 in yeast, Bcmdl1 is a typical half-type ABC transporter localized in the mitochondria of *B. cinerea*. Since Bcmdl1 is an ABC transporter, the expression level might contribute to its function. Therefore, *Bcmdl1* was overexpressed by replacing its original promoter with the promoter of the *EF-1* gene. Unfortunately, although the expression level of *Bcmdl1* was significantly increased in some positive transformants, the transformants were as sensitive as the parental isolate to cyprodinil. Such failures were also experienced using S. cerevisiae (38). Furthermore, the expression level of Mdl1 in yeast always stayed low, even under various environmental stresses (42). Consistent with the above-mentioned results, the expression level of *Bcmdl1* was slightly altered in both the sensitive and resistant isolates after treatment with cyprodinil. Generally, the genes encoding efflux transporters could be hundreds-of-times upregulated in multidrug-resistant (MDR) strains (13). Because of the low expression level, *Bcmdl1* could not be related to MDR, which was also supported by a fungicide sensitivity test where the knockout of *Bcmdl1* or the insertion of the E407K mutation did not alter the fungicide resistance profiles, except for AP resistance, compared to the corresponding parental isolates. In conclusion, *Bcmdl1* was specifically involved in AP resistance, and this resistance was not caused by its overexpression.

Although the function of *Bcmdl1* was not likely related to the expression level, the knockout of *Bcmdl1* resulted in reductions in conidial germination and virulence. Unexpectedly, *Bcmdl1* did not mediate resistance to oxidative stress in *B. cinerea*, which was not consistent with that in yeast (40). The subcellular localization of Bcmdl1 demonstrated that Bcmdl1 was localized in mitochondria, as expected. The decreased content of ATP in the knockout transformants of *Bcmdl1* after cyprodinil treatment indicated that *Bcmdl1* was related to ATP synthesis, as reported previously for yeast (41). Decreased ATP production might lead to declines in conidial germination and virulence in the knockout transformants of *Bcmdl1*, and further research is needed. Genes that affected conidial germination and virulence through energy metabolism were also reported previously in Beauveria bassiana and Magnaporthe oryzae (43, 44). Furthermore, these results also suggested that AP fungicides might target ATP synthase, thereby interfering with energy metabolism. No significant difference between the knockout transformants and the parental isolate in the MP indicated that Bcmdl1 had no effect on the proton gradient at two sides of the membrane. Therefore, it was more likely that *Bcmdl1* was involved in ATP synthesis by regulating the activity of the ATP synthase, as reported previously for yeast (41). Moreover, no significant difference was observed between the various transformants and the corresponding parental isolates for the other contents, including NAD^+^/NADH, MDA, ROS, and H_2_O_2_, illustrating that the regulation of ATP synthesis was the specific function of *Bcmdl1* on mitochondria under treatment with cyprodinil.

**FIG 6:**
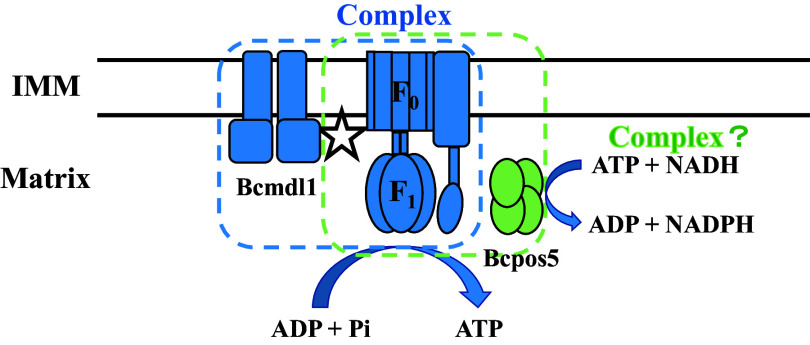
Proposed model for ATP synthase as the primary target of AP fungicides. Bcmdl1 and ATP synthase could form a complex, thereby interfering with ATP production. Bcpos5 might also interact with ATP synthase, but more research is needed. IMM, inner mitochondrial membrane. The possible target site of AP fungicides is marked with a white star. This model is based on one described previously by Mosbach et al. (7).

In a recent study, mutations within nine individual genes involved in mitochondrial processes were determined to confer resistance to AP fungicides in *B. cinerea*, suggesting that AP fungicides primarily target the mitochondria (7). Among all of the mutations, L412F in *Bcpos5* was detected in 55% of the tested resistant field isolates, while E407K in *Bcmdl1* was detected in only 10% of the tested resistant field isolates (7). However, in the current study, the frequency of the resistance mutation E407K in *Bcmdl1* was nearly 65%, while the L412F mutation in *Bcpos5* was detected at a frequency of only 25% (see Table S2 in the supplemental material), demonstrating that different places might have different major mutations, and the E407K mutation in *Bcmdl1* was the major mechanism of AP resistance in *B. cinerea* in Hubei Province, China. Altogether, the frequencies of mutations E407K and L412F were almost 90% in this study (Table S2) and 70% in Europe (7), indicating that these two mutations were the major mechanisms of AP resistance worldwide. According to a previous study, the level of resistance conferred by mutation E407K in *Bcmdl1* was significantly higher than that conferred by mutation L412F in *Bcpos5*, but the combination of E407K and L412F mutations conferred levels of resistance similar to those conferred by the E407K mutation alone (7). Pos5 is an NADH kinase responsible for the production of mitochondrial NADPH, which is a reductant and plays an important role in ROS detoxification in S. cerevisiae (22, 23). Pos5 is also essential for efficient iron-sulfur cluster biogenesis (45). Given the multiple biochemical pathways of Pos5 and the substrates or products of many other resistance genes, *Bcpos5* was considered a target of AP fungicides (7). However, the deletion of Pos5 is not lethal, and there are other NADH kinases in yeast (46), so it is not likely to be a primary target of fungicides. Based on this study, we hypothesize that ATP synthase was a potential target of AP fungicides for the following reasons (Fig. 6). First, *Bcmdl1* was specifically involved in AP resistance. Second, the E407K mutation corresponded to position E332 of Mdl1 in yeast, occurring in the transmembrane domain helices. This position was predicted to face the interior of the channel and may form part of the substrate-binding cavity (47). Since Bcmdl1 was not involved in MDR, we supposed that E407 is the key position where Bcmdl1 may interact with the synthase (41). Third, almost all of the resistance genes identified in recent studies were related to mitochondrial processes. Above all, AP fungicides interfered with the production of ATP. Therefore, it is reasonable that AP resistance-associated Bcmdl1 could form a complex with ATP synthase, and AP fungicides might target the interface between Bcmdl1 and ATP synthase. If there is a mutation in the *Bcmdl1* gene, it may alter the conformation of the complex and reduce the affinity for AP fungicides, thereby leading to resistance. However, more experiments are required to test this hypothesis. This study significantly contributes to the elucidation of the mechanism of resistance to and mode of action of AP fungicides, which will also be beneficial for resistance monitoring and management.

## MATERIALS AND METHODS

### Fungal isolates.

Isolates HBTom-400 and HBTom-103 were used to conduct the sexual cross. They were collected from greenhouse tomatoes in Hubei Province, China. Isolate HBTom-400 was resistant to cyprodinil, while isolate HBTom-103 was sensitive, which had been determined in a previous study (33). In order to confirm the SNPs found by QTL-seq, 109 other resistant isolates and 35 other sensitive isolates collected from greenhouse tomatoes and strawberries in Hubei Province were also tested. All isolates were cultured on potato dextrose agar (PDA) plates (200 g of potato, 20 g of dextrose, and 15 g of agar per L) at 20°C in the dark.

### Sexual cross.

Isolates HBTom-400 and HBTom-103 were used to perform the sexual cross. Before the sexual cross, their mating types were identified by using a PCR method (48). A 930-bp fragment obtained in the PCR product indicated mating type I, while a 750-bp fragment indicated mating type II. According to this method, HBTom-400 was identified as a mating type I isolate, and HBTom-103 was identified as a mating type II isolate. The sexual cross was performed basically according to a previously described method (49). Both isolates were cultured on PDA for 4 weeks at 15°C in the dark, and they were then incubated at 0°C for 4 weeks to induce the formation of sclerotia and microconidia, respectively. Sclerotia were collected and brushed in sterile water using a toothbrush to separate them from agar debris and macroconidia. Microconidia were collected by scraping the colony surface into 20 mL of sterile water using a glass spreading rod. The cross was conducted in a six-well microtiter plate, and approximately three sclerotia and 3 mL of the microconidial suspension were mixed in each well. The plate was incubated at 12°C with a 12-h photoperiod for ~2 months until apothecia developed. When the apothecia turned brown, they were collected and washed with 0.01% Tween 20 three times. Later, the apothecia were crushed using a glass rod in sterile water to release the ascospores, and the ascospores were then isolated by single-spore-separation microscopy (Wuhan Heipu Science and Technology, Ltd., Wuhan, China).

### Fungicide sensitivity test.

To evaluate the resistance of the selected isolates and ascospore progenies to cyprodinil, mycelial plugs (5 mm in diameter) cut with a cork borer from the margin of a 3-day-old colony of each isolate were transferred upside down onto Czapek-Dox agar (CzA) (2 g NaNO_3_, 0.5 g KCl, 0.5 g MgSO_4_·7H_2_O, 0.01 g Fe_2_SO_4_, 1 g K_2_HPO_4_, 0.01 g ZnSO_4_, 0.005 g CuSO_4_·5H_2_O, 30 g sucrose, and 15 g agar per L) plates containing 10 μg/mL cyprodinil. The plates were incubated at 20°C in the dark for 3 days, and the colony diameter was then observed visually. Isolates able to grow on the cyprodinil-amended medium were considered resistant isolates, while isolates unable to grow were considered sensitive ones.

The sensitivity of the selected isolates to cyprodinil was determined by a mycelial growth assay. Mycelial plugs (5 mm in diameter) were cut from the edge of a 3-day-old colony and placed upside down on CzA plates amended with cyprodinil at 0, 0.3, 1, 3, 10, 30, or 100 μg a.i. (active ingredient)/mL. After 3 days of incubation at 20°C, the colony diameters were determined by calculating the means of two perpendicular colony diameters. The percentage of mycelial growth inhibition was calculated according to the formula percent inhibition = [1 − (mean colony diameter on treated plates − plug diameter)/(mean colony diameter on untreated plates − plug diameter)] × 100%. The EC_50_ value, representing an assessed concentration at which mycelial growth is inhibited by 50%, was calculated by regressing the percentages of mycelial growth inhibition against the log_10_ of the fungicide concentrations.

Besides cyprodinil, the resistance of the selected transformants to other fungicides was also examined. Resistance to the MBC fungicide carbendazim, the DCF fungicide iprodione, the QoI fungicide azoxystrobin, and the PP fungicide fludioxonil was investigated using PDA plates containing the corresponding fungicides at 1, 5, 10, and 0.4 μg/mL, respectively, and resistance to the SDHI fungicide boscalid was determined using yeast Bacto acetate (YBA) (10 g yeast extract, 10 g Bacto peptone, and 20 g sodium acetate per L) agar plates amended with 75 μg/mL boscalid. Fresh PDA plates inoculated with mycelial plugs were used as controls. After 3 days of incubation at 20°C in the dark, mycelial growth was observed visually. Isolates that could grow on fungicide-amended plates were considered resistant isolates.

### DNA extraction.

Three different DNA extraction methods were adopted in this study according to the purposes. Due to the need for high-quality DNA for genome resequencing, the genomic DNA was extracted using a sodium laurate method (50). Briefly, mycelia harvested from 3-day-old colonies on PDA were ground to a powder with a pestle in liquid nitrogen. Afterward, the genomic DNA was released with sodium laurate and purified by phenol-chloroform extraction and isopropanol-ethanol precipitation. The DNA was resolved in double-distilled water (ddH_2_O), and RNA was removed by coincubation with RNase A (TransGen, Beijing, China) for 1 h. A quick and simple method was applied to extract the DNA for PCR and Sanger sequencing to confirm the potential resistance-related SNPs (51). The isolates were first cultured on PDA plates with a cellophane membrane. Subsequently, the mycelia were collected, suspended in 10× Tris-EDTA (TE) (100 mM Tris-HCl, 10 mM EDTA [pH 8.0]), and bathed in boiling water for 2 min. After centrifugation, the supernatant was used as the DNA solution. The DNA of the transformants was extracted by a quick and safe method (52). The mycelia of the transformants were collected after 3 days of incubation on PDA plates with a cellophane membrane and broken down in extraction buffer (1 M KCl, 100 mM Tris-HCl, 10 mM EDTA) using pestles. The DNA was then precipitated with isopropanol-ethanol and resolved in ddH_2_O. All of the DNA solutions were stored at −20°C until further use.

### PCR.

PCR was conducted using a thermal cycler (Eastwin, Suzhou, China). For DNA constructs for transformation, a 50-μL volume containing 1× Phanta max buffer, 1 μL deoxynucleoside triphosphate (dNTP) mix (10 mM each), 2 μL each forward and reverse primers (10 μM), 1 μL Phanta max superfidelity DNA polymerase (Vazyme, Nanjing, China), and 2 μL template DNA (100 ng/μL) was used. PCR master mix (Yeasen, Shanghai, China) was selected for gene amplification, Sanger sequencing, and other purposes. The reaction was performed in a volume of 25 μL containing 1× PCR master mix, 1 μL each forward and reverse primers (10 μM), and 1 μL template DNA (100 ng/μL). Conditions for PCR constituted an initial preheating step at 94°C for 3 min followed by 35 cycles of denaturation at 94°C for 30 s, annealing at 58°C for 30 s, and extension at 72°C for 30 s, with a final extension step at 72°C for 10 min. The PCR products were analyzed by electrophoresis on a 1% agarose gel stained with Golden View (Aidlab, Beijing, China).

### RNA extraction and cDNA synthesis.

TriPure reagent (Aidlab) was used to extract the total RNA. After incubation with shaking in potato dextrose broth (PDB) (200 g of potato and 20 g of dextrose per L) at 140 rpm at 20°C for 3 days, the mycelia of the selected isolates were collected by filtration through two layers of cheesecloth and ground into a powder in liquid nitrogen. Subsequently, the total RNA was released using TriPure reagent and purified by chloroform extraction and ethanol precipitation. The RNA was resolved in ddH_2_O and stored at −80°C. The 1st-strand cDNA was synthesized using Hifair II 1st-strand cDNA synthesis supermix (Yeasen). To remove the residual DNA, 3 μg RNA was mixed gently with 2 μL 5× gDNA digester buffer and 1 μL gDNA digester in a volume of 10 μL. After incubation at 42°C for 2 min, 10 μL 2× Hifair II supermix was added to the system, and the mixture was incubated at 25°C for 5 min followed by 42°C for 30 min and, finally, 85°C for 5 min to inactivate the enzyme. The cDNA was maintained at −20°C and diluted six times before use.

### Reverse transcription-quantitative PCR.

Quantitative PCR (qPCR) was performed using a volume of 10 μL containing 1× Sybr green qPCR mix (Aidlab), 0.5 μL each forward and reverse primers (10 μM), and 1 μL template cDNA. To determine the relative expression levels of the target genes, the β-tubulin gene *BctubA* was chosen as the internal reference gene, which was partially amplified by using primer pair qTubA-F/qTubA-R (see Table S5 in the supplemental material). Primer pair q820-1640F20/q820-1771R19 was used to amplify the partial sequence of the *Bcmdl1* gene. A QuantStudio 7 Flex real-time PCR system (Applied Biosystems, CA, USA) was used to perform qPCR under the following conditions: an initial preheating step at 94°C for 3 min followed by 40 cycles of denaturation at 95°C for 20 s, annealing at 56°C for 20 s, and extension at 72°C for 20 s. The fluorescence signal was detected at the extension stage of each cycle. A melt curve was obtained by incubating the products at 94°C for 20 s, 65°C for 1 min, and 95°C for 15 s. When increasing the temperature from 65°C to 95°C at a rate of 0.05°C/s, the fluorescence signal was detected simultaneously. The expression level of the *Bcmdl1* gene was normalized to the expression level of the *BctubA* gene, and relative gene expression was calculated by the comparative threshold cycle (*C_T_*) (2^−ΔΔ^*^CT^*) method (53). The experiment was performed in triplicate and repeated twice.

### Genome resequencing and data analysis.

To prepare the resistant pool and the sensitive pool, DNAs from seven cyprodinil-resistant progenies and six cyprodinil-sensitive progenies were mixed at equal ratios, respectively. Along with the DNA of the parental isolates, the bulk DNAs of the resistant pool and the sensitive pool were sent to Wuhan Unique Bio-information Company to perform genome resequencing using an Illumina genome analyzer.

Reads containing adapters, >10% unknown nucleotides (N’s), or >50% low-quality (*Q* value of ≤20) bases were filtered to obtain high-quality clean reads before data analysis. The clean reads from each sample were further aligned against the public reference genome (https://www.broadinstitute.org/fungal-genome-initiative/botrytis-cinerea-genome-project) with the settings mem 4 -k 32 -M using Burrows-Wheeler Aligner (54). Alignment files were converted to SAM/BAM files by using SAMtools (55). Variant calling was performed using the Genome Analysis Toolkit (GATK) unified genotyper (56). SNPs were filtered using GATK’s variant filtration with the proper standards (-Window 4, -filter “QD < 4.0 ‖ FS > 60.0 ‖ MQ < 40.0,” -G_filter “GQ < 20”), and those exhibiting segregation distortion or sequencing errors were discarded. In order to determine the physical positions of each variant, the ANNOVAR software tool was used to align and annotate SNPs or insertions-deletions (indels) (57). Sliding-window analysis was applied to the frequency distribution of SNPs (SNP index) in the population of bulk individuals, and the SNP index was calculated for all of the SNP positions. SNP positions with an SNP index of <0.3 or >0.7 and a read depth of <7 from the two sequences were excluded, as these may represent spurious SNPs called due to sequencing and/or alignment errors. The Δ(SNP index) value was calculated by the subtraction of the SNP index between two bulk pools. QTLs were identified in these positive or negative peak regions with 95% confidence intervals using 10,000 bootstrap replicates. SNPs in the peak regions were selected and annotated to identify potential functional variants.

### Identification of resistance-related SNPs.

According to a previous study, resistance to AP fungicides could be conferred by a mutation in one major gene (6). Based on this hypothesis, resistance-related SNPs were identified according to the following rules. First, the SNPs should be nonsynonymous mutations. Second, the SNPs can be found only in the parental isolate with resistance and the resistant pool. The resistance-related SNPs were further confirmed by sequencing the same loci with the candidate SNPs from field isolates. If a candidate SNP was detected in sensitive isolates, then it should be a false positive. Therefore, 109 resistant and 35 sensitive field isolates were selected to perform sequencing identification. The amplification of the DNA fragment was conducted by PCR, and the forward and reverse primers (F-seq/R-seq) were designed 300 bp upstream and downstream of the resistance-related SNPs, respectively, using Oligo7 (Molecular Biology Insights, CO, USA) (Table S5). Furthermore, the frequency of mutation L412F in *Bcpos5* was also investigated using primer pair 2880-1030F21/2880-1555R21. This mutation was widely detected in resistant field isolates in Europe and was considered to be a good candidate as the primary target of AP fungicides (7). After analysis by electrophoresis, the PCR products were sequenced at Wuhan Tianyi Huiyuan Biotechnology Co., Ltd.

### DNA constructs for transformation.

Constructs for the validation of the candidate resistance-related SNPs were amplified by PCR. Approximately 500-bp fragments, including both flanking regions, of candidate SNPs were amplified with the corresponding F-snp/R-snp primer pairs using the DNA of resistant isolate HBTom-400 as the template (Table SS). After purification using an EasyPure PCR purification kit (TransGen), the constructs were stored at −20°C until transformation.

Constructs for other transformations (E407K insertion, knockout, complementation, overexpression, and labeling) were obtained by double-joint PCR (58). In the first round of PCR, the upstream and downstream regions of the candidate gene were amplified with primer pairs F1/R1 and F2/R2, respectively. Also, hygromycin B resistance gene *hph* (hygromycin B phosphotransferase) in vector pSKH and neomycin resistance gene *neo* (amino 3’-glycosyl phosphotransferase) in vector pCETNS-4 were amplified with primer pairs Hyg-F/R and Neo-F/R, respectively. After purification using an EasyPure PCR purification kit (TransGen), a second round of PCR was conducted to fuse the upstream and downstream fragments with *hph* or *neo*, respectively. To increase the accuracy of homologous recombination, constructs with the split marker were produced by a third round of PCR (59). The upstream and downstream regions of the fused fragments were amplified using primer pairs F3/HY-R or F3/NE-R and YG-F/R3 or EO-F/R3, respectively. Finally, the products from the third round of PCR were purified using an E.Z.N.A. gel extraction kit (Omega, Norcross, GA, USA) and used for transformation.

To overexpress the *Bcmdl1* gene, the upstream promoter region of the *EF-1* gene was amplified with primer pair F1ef/R1ef and fused with both the *hph* gene and the *Bcmdl1* coding sequence by double-joint PCR. The final PCR product was used as the template to amplify the downstream construct with primer pair YG-F/R3, and the upstream construct was prepared with primer pair F3/HY-R for overexpression transformation. To label the Bcmdl1 protein with green fluorescent protein (GFP), the vector pCB-GFP-Bcmdl1 was constructed. First, the *Bcmdl1* gene without a stop codon was amplified with primer pair z820-2219F21/z820-4646R20 and purified by using an EasyPure PCR purification kit (TransGen). Next, the vector pCB-GFP was digested with the endonucleases EcoRV (TaKaRa, Dalian, China) and SmaI (TaKaRa). After purification by using an EasyPure PCR purification kit (TransGen), the linearized vector was recombined with the *Bcmdl1* gene using a ClonExpress II one-step cloning kit (Vazyme) according to the manufacturer’s instructions. The recombined plasmid was validated by colony PCR with primer pair GFP-ceF/GFP-ceR and Sanger sequencing. The upstream construct for GFP transformation was amplified using the pCB-GFP-Bcmdl1 vector as the template with primer pair 820-ATG/NE-R, and the downstream construct was prepared with primer pair EO-F/R3 as described above.

### Transformation of *B. cinerea*.

The transformation of *B. cinerea* was achieved by PEG-mediated protoplast transformation. First, the isolate was incubated in 50 mL PDB for 2 days at 140 rpm at 20°C. Mycelial balls were then collected, crushed to flocculence, and transferred to 150 mL yeast extract-peptone-dextrose (YEPD) medium (3 g of yeast extract, 10 g of peptone, and 20 g of dextrose per L [pH 7.5]) for another day of incubation at 140 rpm at 20°C. The cultures were then centrifuged at 8,000 rpm for 5 min to gather the mycelia. The mycelia were washed with a 0.6 M KCl solution (0.6 M KCl and 100 mM CaCl_2_) once and resuspended in 0.6 M KCl containing 0.01 g/mL cell wall lyase (lysing enzymes from Trichoderma harzianum; Sigma). After shaking at 25°C at 100 rpm for 3.5 h, protoplasts were filtered through six layers of cheesecloth, centrifuged for 5 min at 4°C at 2,000 × *g*, and washed in ice-cold STC buffer (1.2 M sorbitol, 10 mM Tris-HCl [pH 7.5], 50 mM CaCl_2_). The protoplasts were then resuspended in ice-cold STC buffer, and the concentration was adjusted to 10^8^ protoplasts/mL with the aid of a hemacytometer. To perform the transformation, 200 μL of protoplasts was mixed gently with 40 μL of transformation constructs, and the mixture was placed on ice for 15 min. Next, 200 μL SPTC buffer (STC buffer plus 40% PEG 4000) was gently added to the mixture. After incubation on ice for 15 min, 200 μL, 200 μL, and 600 μL SPTC were added successively to the mixture, and the mixture was incubated at 20°C for 20 min. Finally, the mixture was transferred to 20 mL sucrose-HEPES (SH) medium (0.6 M sucrose and 5 mM HEPES) and incubated for 40 min at 20°C, followed by incubation overnight at 20°C at 100 rpm. When the protoplasts germinated and snowflakes appeared, the culture was mixed with CzA containing cyprodinil or PDA amended with the corresponding antibiotics and poured into a petri dish. After several days of incubation, the transformant that grew enough to form a colony was transferred onto CzA plates with cyprodinil or PDA plates with the corresponding antibiotics for two generations. The remaining transformants were considered candidates for positive transformants and were used for further confirmation.

### Transformant verification and purification.

PCR analysis was performed for the further confirmation of transformants. The integration of the *hph* or *neo* cassette into transformants was verified by PCR using primer pair Hyg-GF/Hyg-GR or Neo-GF/Neo-GR. The left or right flanking regions of the homologous fragment were also examined using primer pairs F1/Hyg-CR or F1/Neo-CR and Hyg-CF/R2 or Neo-CF/R2. For GFP transformants, the left flanking region was confirmed using primer pair 820-1881F21/Neo-CR. Since *B. cinerea* is a classic multinucleate fungus, whether transformants were homozygotes or heterozygotes was also analyzed by using primer pair F4/R4. Usually, the PCR products with primer pair F4/R4 contained two fragments. One of them indicated the successful transformation of DNA constructs, and the other one suggested the existence of wild nucleus. To improve homozygosis, the transformants were cultured on PDA containing 100 μg/mL of the corresponding antibiotics until sporulation, and a single spore was then isolated. After 3 days of cultivation on PDA, the DNA of these transformants was extracted, and their homozygosis was identified by PCR as described above.

### Bioinformatics analysis of *Bcmdl1*.

The nucleotide and amino acid sequences were downloaded from EnsemblFungi (http://fungi.ensembl.org/Botrytis_cinerea/Info/Index). The full length of the *Bcmdl1* fragment was amplified with primer pair 820-ATG/820-TAG (Table S5) using gDNA and cDNA as the templates, respectively. Based on the comparison of these two sequences, the exons and introns of *Bcmdl1* were identified. The BLASTP program was used to determine the amino acid sequence of Bcmdl1 to search for its homologous proteins in the National Center for Biotechnology Information (NCBI) database. Phylogenetic analysis of Mdl1 proteins was conducted using amino acid sequences, and a phylogenetic tree was generated with MEGA 7 software using the maximum likelihood method. The functional domains of Bcmdl1 were predicted using SMART (Simple Modular Architecture Research Tool) (http://smart.embl-heidelberg.de/).

### Phenotypes of knockout transformants.

In addition to fungicide resistance, the function of *Bcmdl1* was also analyzed by determining the various phenotypes of the knockout transformants. Mycelial growth was measured on fresh PDA plates, and sensitivity to NaCl, sorbitol, SDS, hydrogen peroxide (H_2_O_2_), and CR was determined using PDA plates amended with the corresponding chemicals at 0.5 M, 1.2 M, 0.01% (wt/vol), 5 mM, and 300 μg/mL, respectively. The plates were incubated at 20°C in the dark for 3 days, and the colony diameter was determined by calculating the mean of two perpendicular diameters. The percentage of mycelial growth inhibition was calculated as described above for the sensitivity test. Acid production was examined on PDA plates containing 0.005% (wt/vol) bromophenol blue. After 3 days of incubation, the colony diameter and the diameter of the yellow halo around the fungal colony were measured, and the ratio of the mycelial diameter to the yellow halo diameter was then calculated. After the colony diameter was measured, the plates used for mycelial growth were maintained for another 3 weeks to induce sporulation and sclerotium production. Conidia were collected by rinsing the plates with 3 mL sterilized water using a cotton swab, and the conidial suspension was filtered through a piece of lens cleaning paper. The conidial concentration was determined with the aid of a hemacytometer. Conidial germination was analyzed on 1.5% water agar after incubation for 6, 9, 12, and 24 h, and 100 conidia were randomly selected. Subsequently, the number of sclerotia was recorded visually, and ~20 sclerotia were randomly collected. After sterilization with 70% (vol/vol) ethanol and rinsing with sterile water, the dried sclerotia were stored at 4°C for 3 months. The viability of the sclerotia was determined by calculating the percentage of germinated sclerotia on fresh PDA plates after 3 days of incubation against all sclerotia. Finally, tomato fruit inoculated with a 10-μL conidial suspension at a concentration of 2 × 10^5^ spores/mL was used to investigate virulence. After incubation at room temperature (~25°C) for 5 days, the lesion size was measured. Data for each phenotype were subjected to one-way analysis of variance (ANOVA). Mean values among different groups were compared using the least significant difference (LSD) test at a *P* value of 0.05. Percentage data and sporulation data were arcsine and logarithm transformed, respectively, before analysis. The statistical analyses were performed using SPSS Statistics (version 19.0; IBM Corporation, USA). This experiment had three replicates and was repeated twice.

### Subcellular localization of Bcmdl1.

The subcellular localization of Bcmdl1 tagged with GFP was examined using a Leica (Wetzlar, Germany) TCS SP8 confocal microscope. For the observation of Bcmdl1::GFP, mycelial balls of the GFP transformant obtained from a 2-day incubation in PDB were crushed to flocculence and transferred to YEPD medium for another day of incubation. To mark the mitochondria, the mycelia were stained with Mito-Tracker red CMXRos (Beyotime, Shanghai, China) according to the manufacturer’s instructions. The fluorescence signals of GFP and Mito-Tracker dye were then examined, and the images were processed using Leica LAS X software.

### Analysis of mitochondrial function in knockout and insertion transformants.

The mitochondrial function of three transformants for each knockout and E407K insertion was analyzed by investigating several parameters with or without treatment with cyprodinil. The content of ATP was measured by the luminescence produced by the conversion of luciferin into oxyluciferin, which was catalyzed by firefly luciferase consuming ATP (ATP assay kit; Beyotime). The content of NAD^+^/NADH was analyzed by the color development reaction based on water-soluble tetrazolium salts (WST-8) (NAD^+^/NADH assay kit with WST-8; Beyotime). The production of ROS was indicated by the fluorescence probe 2′,7′-dichlorofluorescein diacetate (DCFH-DA), which produced green-like fluorescence by ROS after several cellular processes (reactive oxygen species assay kit; Beyotime). The contents of malondialdehyde (MDA) and H_2_O_2_ were investigated by a color development reaction based on thiobarbituric acid and titanium(IV) sulfate, respectively (MDA assay kit and H_2_O_2_ content assay kit; Boxbio). The MP was visualized by using the fluorescence probe JC-1, which shows red fluorescence when the MP is high (mitochondrial membrane potential assay kit with JC-1; Beyotime). All of the experiments were performed according to the manufacturers’ instructions, and the mycelia used for analysis were prepared as described above for the subcellular localization analysis.
