# Supplementary material for: Mitochondrial Inner Membrane ABC Transporter Bcmdl1 Is Involved in Conidial Germination, Virulence, and Resistance to Anilinopyrimidine Fungicides in Botrytis cinerea
Source: Microbiol Spectr. 2023 Jun 15;11(4):e00108-23. doi: 10.1128/spectrum.00108-23 (PMC10434148; doi:10.1128/spectrum.00108-23)
Supplement: Supplemental file 1 — Supplemental material. Download spectrum.00108-23-s0001.pdf, PDF file, 1.6 MB [file spectrum.00108-23-s0001.pdf]

Supporting information

Table S1 Screening and annotation of candidate SNPs

	Gene	No. of SNPs	Gene Function
	Bcin09g05000	1	similar to N-acetyltransferase
	Bcin09g05010	2	similar to fasciclin domain family protein
	Bcin09g05340	1	bikaverin cluster-transcription factor
	Bcin09g05420	2	hypothetical protein BofuT4_P066430.1
	**Bcin16g00820**	3	similar to ABC transporter
	**Bcin16g00900**	1	predicted protein
	**Bcin16g00950**	4	carbohydrate-Binding Module family 18 protein
	Bcin16g00970	1	P-loop containing nucleotide triphosphate hydrolase protein
	**Bcin16g00980**	4	similar to Hsp70 family protein
	Bcin16g00990	9	similar to transcription factor Cys6
	Bcin16g01010	1	hemolysin-III channel protein Izh2
Total	11	29	

Genes in bold are the final four candidate genes responsible for anilinopyrimidine resistance.

Table S2 Confirmation of resistance related SNP by sequencing in anilinopyrimidine resistant isolates

Isolates[a]				Mutations[b]								
Name	Crop	Location	Year	E407K	D780N	M94I	S525I	A995V	I71V	E359D	L412F	E407K + L412F
HBStr-55	Strawberry	Wuhan	2012	0	0	0	0	0	0	0	0	0
HBStr-76	Strawberry	Wuhan	2012	0		0					1	1
HBStr-78	Strawberry	Wuhan	2012	1	0	1	0	0	0	0	0	1
HBStr-79	Strawberry	Wuhan	2012	1	0	1	0	0	0	0	0	1
HBStr-82	Strawberry	Wuhan	2012	1	0	1	0	0	0	0	0	1
HBStr-85	Strawberry	Wuhan	2012	1	0	1	0		0	0	0	1
HBStr-87	Strawberry	Wuhan	2012	1		1						1
HBStr-94	Strawberry	Wuhan	2012	1							0	1
HBStr-100	Strawberry	Wuhan	2012	0		0					1	1
HBStr-102	Strawberry	Wuhan	2012	1		1		0	0	0	0	1
HBStr-106	Strawberry	Wuhan	2012	1		1					0	1
HBStr-110	Strawberry	Wuhan	2012	0							0	0
HBStr-111	Strawberry	Wuhan	2012	1	0	1	0	0		0	0	1
HBStr-116	Strawberry	Wuhan	2012	0	0	1	1	1	0	0	1	1
HBStr-117	Strawberry	Wuhan	2012	0			0				1	1
HBStr-120	Strawberry	Wuhan	2012	0	0	0	0	0	0	0	0	0
HBStr-125	Strawberry	Yichang	2012	0							0	0
HBStr-128	Strawberry	Yichang	2012	1	0	1	0	0	0	0	0	1
HBStr-132	Strawberry	Yichang	2012	0	0	0	0		0	0	1	1
HBStr-133	Strawberry	Yichang	2012	1	1	1	1	1	1	1	0	1
HBStr-155	Strawberry	Yichang	2012	1	1	1	1	1	1	1	0	1
HBStr-174	Strawberry	Xiaogan	2012	0	0	0	0	0	0	0	0	0
HBStr-177	Strawberry	Xiaogan	2012	0		0	0		0	0	0	0
HBStr-179	Strawberry	Xiaogan	2012	0		0					0	0
HBStr-183	Strawberry	Xiaogan	2012	1	0	0	0		0		0	1

HBStr-184	Strawberry	Shiyan	2012	0		0					0	0
HBStr-185	Strawberry	Shiyan	2012	0		0					0	0
HBStr-187	Strawberry	Shiyan	2012	0	0	0	0		0	0	1	1
HBStr-188	Strawberry	Shiyan	2012	0	0	0	0		0	0	1	1
HBStr-189	Strawberry	Shiyan	2012	0		0					0	0
HBStr-191	Strawberry	Shiyan	2012	1							0	1
HBStr-196	Strawberry	Shiyan	2012	1							0	1
HBStr-241	Strawberry	Yichang	2012	1		1					0	1
HBStr-277	Strawberry	Suizhou	2013	1		1					0	1
HBStr-278	Strawberry	Suizhou	2013	0		1					1	1
HBStr-280	Strawberry	Suizhou	2013	1		1					0	1
HBStr-285	Strawberry	Suizhou	2013	1		1					0	1
HBStr-289	Strawberry	Suizhou	2013	1		1					0	1
HBStr-295	Strawberry	Xiantao	2013	0	0	1	1	1	0	0	0	0
HBStr-298	Strawberry	Yichang	2013	1							0	1
HBStr-299	Strawberry	Yichang	2013	0		0					0	0
HBStr-301	Strawberry	Xiaogan	2013	1	1	1	1	1	1	1		1
HBStr-302	Strawberry	Xiantao	2013	1		1					0	1
HBStr-317	Strawberry	Yichang	2013	1	1	1	1	1	1	1	0	1
HBStr-334	Strawberry	Yichang	2013	0							1	1
HBStr-348	Strawberry	Xiantao	2013	1	0	1	0	0	0	0	0	1
HBStr-383	Strawberry	Xianning	2013	0	0	1	0	0	0	0	0	0
HBStr-453	Strawberry	Yichang	2013	0		0					0	0
HBStr-458	Strawberry	Yichang	2013	0	0	0	0		0	0	1	1
HBTom-98	Tomato	Qianjiang	2012	1	0	1	0	0	0	0	0	1
HBTom-104	Tomato	Qianjiang	2012	1	0	1	0	0	0	0	0	1
HBTom-106	Tomato	Qianjiang	2012	1	1	1	1	1	1	1	0	1
HBTom-107	Tomato	Qianjiang	2012	1		1	1	1			0	1
HBTom-109	Tomato	Qianjiang	2012	1	1	1	1	1	1	1	0	1
HBTom-121	Tomato	Qianjiang	2012	1		1					0	1

HBTom-128	Tomato	Qianjiang	2012	1	1	1	1	1	1	1	0	1
HBTom-131	Tomato	Qianjiang	2012	1	1	1	1	1	1	1	0	1
HBTom-135	Tomato	Qianjiang	2012	1	1	1	1	1	1	1	0	1
HBTom-185	Tomato	Jingzhou	2012	1		0					0	1
HBTom-229	Tomato	Shiyan	2012	1		1					0	1
HBTom-316	Tomato	Huanggan	2013	1	0	0	0		0		0	1
HBTom-356	Tomato	Jingmen	2013	1	1	1	1	1	1	1	0	1
HBTom-358	Tomato	Jingmen	2013	1	1	1	1	1	1	1	0	1
HBTom-361	Tomato	Xiangyan	2013	0		0					1	1
HBTom-364	Tomato	Jingmen	2013	1		1					0	1
HBTom-368	Tomato	Jingmen	2013	1							1	2
HBTom-370	Tomato	Jingmen	2013	1	1	1		1	1	1	0	1
HBTom-371	Tomato	Jingmen	2013	0	0	0	0		0	0	1	1
HBTom-373	Tomato	Jingmen	2013	1		1	1	1	1	1	0	1
HBTom-374	Tomato	Jingmen	2013	1	1	1	1	1	1	1	0	1
HBTom-377	Tomato	Jingmen	2013	1	1	1	1	1	1	1	0	1
HBTom-378	Tomato	Jingmen	2013	0	0	0	0		0	0	1	1
HBTom-379	Tomato	Jingmen	2013	1	1	1	1	1	1	1	0	1
HBTom-381	Tomato	Jingmen	2013	0	0	0	0		0	0	1	1
HBTom-383	Tomato	Jingmen	2013	1		1	1	1	1	1	0	1
HBTom-385	Tomato	Jingmen	2013	1	1	1	1	1	1	1	0	1
HBTom-386	Tomato	Jingmen	2013	0		0					1	1
HBTom-387	Tomato	Jingmen	2013	1	1	1	1	1	1	1	0	1
HBTom-388	Tomato	Jingmen	2013	1	1	1	1	1	1	1	0	1
HBTom-390	Tomato	Jingmen	2013	1	1	1	1	1	1	1	0	1
HBTom-391	Tomato	Jingmen	2013	1	1	1	1	1	1	1	0	1
HBTom-392	Tomato	Jingmen	2013	1	1	1	1	1	1	1	0	1
HBTom-394	Tomato	Jingmen	2013	1		1					0	1
HBTom-395	Tomato	Jingmen	2013	1	1	1	1	1	1	1	0	1
HBTom-396	Tomato	Jingmen	2013	1	1	1	1	1	1	1	0	1

HBTom-397	Tomato	Jingmen	2013	1	1	1	1	1	1	1	0	1
HBTom-398	Tomato	Jingmen	2013	1		1					0	1
HBTom-399	Tomato	Jingmen	2013	1		1					0	1
HBTom-400	Tomato	Jingmen	2013	1		1					0	1
HBTom-401	Tomato	Jingmen	2013	1		1					0	1
HBTom-402	Tomato	Jingmen	2013	0		0					1	1
HBTom-403	Tomato	Jingmen	2013	1		1					0	1
HBTom-404	Tomato	Jingmen	2013	1	1		1	1	1	1	0	1
HBTom-405	Tomato	Jingmen	2013	1		1					0	1
HBTom-406	Tomato	Jingmen	2013	0		0					1	1
HBTom-407	Tomato	Jingmen	2013	1	1	1	1	1	1	1	0	1
HBTom-408	Tomato	Jingmen	2013	1	1	1	1	1	1	1	0	1
HBTom-410	Tomato	Jingmen	2013	1		1					0	1
HBTom-411	Tomato	Jingmen	2013	1	1	1	1	1	1	1	0	1
HBTom-511	Tomato	Xiaogan	2013	0		0	0	0	0	0	0	0
HBTom-516	Tomato	Xiaogan	2013	0		0					1	1
HBTom-517	Tomato	Xiaogan	2013	0		0					1	1
HBTom-518	Tomato	Xiaogan	2013	0		0	0		0	0	1	1
HBTom-520	Tomato	Xiaogan	2013	0		0					1	1
HBTom-521	Tomato	Xiaogan	2013	0		0					1	1
HBTom-522	Tomato	Xiaogan	2013	0		0	0		0	0	1	1
HBTom-527	Tomato	Xiaogan	2013	0		0					1	1
HBTom-529	Tomato	Xiaogan	2013	1							1	1
HBTom-530	Tomato	Xiaogan	2013	1							1	1
Total strawberry				51.02%	18.18%	58.54%	25.00%	35.29%	17.39%	17.39%	21.28%	71.43%
Total tomato				75.00%	80.00%	69.64%	74.29%	90.00%	74.29%	76.47%	28.33%	100.00%
Total average				64.22%	53.85%	64.95%	54.24%	70.21%	51.72%	52.63%	25.23%	87.16%

[a] Information of isolates, including names, crops, collecting locations, and isolation years.

[b] E407K and D780N were mutations in *Bcin16g00820 (Bcmdl1)*. M94I was mutation in *Bcin16g00900*. S525I and A995V were mutations in *Bcin16g00950*. I71V and E359D were mutations in *Bcin16g00980*. L412F was mutation in *Bcin10g02880 (Bcpos5)*. E407K + L412F were known mutations conferring resistance to anilinopyrimidine fungicides in *Botrytis cinerea*. One (1) = positive; Zero (0) = negative; Blank = not detected.

Table S3 Confirmation of resistance related SNP by genetic transformation

Transformants[a]	Mutations[b]						
	E407K	D780N	M94I	S525I	A995V	I71V	E359D
Picked	68	40	40	28	35	33	30
Survived	8	1	1	7	6	2	2
Verified	3	0	0	0	0	0	0

[a] Picked = picked randomly for another round of cyprodinil selection at 20 μg/mL; Survived = survived after three rounds of cyprodinil selection at 20 μg/mL; Verified = verified by Sanger sequencing.

[b] E407K and D780N were mutations in *Bcin16g00820 (Bcmdl1)*. M94I was mutation in *Bcin16g00900*. S525I and A995V were mutations in *Bcin16g00950*. I71V and E359D were mutations in *Bcin16g00980*. One = positive; Zero = negative; Blank = not detected.

Table S4 EC_{50} values of various isolates/transformants to cyprodinil

Isolate/Transformant[a]	EC_{50} (μg/mL)[b]
HBTom-103	0.03 ± 0.02
HBTom-400	30.65 ± 5.58
Bcmdl1^{E407K}-1	24.69 ± 3.37
Bcmdl1^{E407K}-12	25.14 ± 3.11
Bcmdl1^{E407K}-67	32.61 ± 0.78
ΔBcmdl1-3	0.01 ± 0.00
ΔBcmdl1-13	0.02 ± 0.02
ΔBcmdl1-37	0.02 ± 0.01
ΔBcmdl1-37C	24.05 ± 1.13
ovBcmdl1-3	0.05 ± 0.02
ovBcmdl1-6	0.05 ± 0.03
ovBcmdl1-16	0.07 ± 0.02

[a] Isolates HBTom-103 and HBTom-400 were sensitive and resistant filed isolates to anilinopyrimidine fungicides. Transformants *Bcmdl1*^{E407K}-1, 12, and 67 were E407K insertion transformants of *Bcmdl1* with HBTom-103 as parental isolate. Transformants Δ*Bcmdl1*-3, 13, and 37 were knockout transformants of *Bcmdl1* with HBTom-400 as parental isolate. Transformant Δ*Bcmdl1*-37C was complemented transformant of *Bcmdl1* with transformant Δ*Bcmdl1*-37 as parental strain. Transformants *OvBcmdl1*-3, 6, and 16 were overexpression transformants with HBTom-103 as parental isolate.

[b] Mean ± standard deviation (SD). EC_{50} value represents an assessed concentration at which mycelial growth is inhibited by 50%.

Table S5 Primers used in this study

Name	Sequence	Description
Mat-F	AGCTTCTGTTGGTGCGAAGT	Identification primers for mate type
Mat1-1-R	GGGCGACTTTTTCAGTCTTC	
Mat1-2-R	ATGTGGGCTGTTGCAGGTTT	
820-1453F21	CGAATAAAAGCTGGAGACACC	F-seq/R-seq primers for *Bcmdl1*
820-2067R21	GAATTGTAGCCAGACTCCGAT	
820-2356F21	TCTTTCGACTATCAAGCGTTC	
820-2999R20	GATATTCTCACGAAGCCCAA	
900-67F22	GTCTAATCCATCAAACGGCTTC	F-seq/R-seq primers for *Bcin16g00900*
900-471R21	TGCCAAAAGACTCAAAGCCAT	
950-1751F21	TCAACTTCTTCTACTCGACCA	F-seq/R-seq primers for *Bcin16g00950*
950-2289R21	TAGAACTAGCTCCAAGGCCAA	
950-3363F19	CTCAACAAGAACACCTGCT	
950-4317R20	GATACAGACGGCGACACCAA	
980-392F21	TAATTTCCTCATCGCTTTGCT	F-seq/R-seq primers for *Bcin16g00980*
980-939R20	AGTAATTTGAACCTTGCCAT	
980-1451F19	ATTGGCGAAACTTACCCTC	
980-2332R19	CGTACCCCTCTATGAGCAG	
820-3082F20	TTCTTTGATGCCAATCGAGT	F-snp/R-snp primers for E407K in *Bcmdl1*
820-4538R20	GATTTCCTCGGACTCTGTGA	
820-3499F21	TCAAGTGGATTCTTTGGCAAC	F-snp/R-snp primers for D780N in *Bcmdl1*
820-5192R20	GCTCGTTTTCCTTCCTCTCG	
900-3625F21	CTCGCTAAATTAACAAGTCCA	F-snp/R-snp primers for M94I in *Bcin16g00900*
900-5016R21	ATTCTATTGTTCTAAGCCCAG	
950-5859F20	GTAGCAACTTTCAGGACATT	F-snp/R-snp primers for S525I in *Bcin16g00950*
950-7788R22	CGCATTAGAATTCATTACGAGA	
950-6526F21	TTCTTCCTCACGCAGAAGCAA	F-snp/R-snp primers for A995V in *Bcin16g00950*
950-8485R21	GAATCATAAGACTCGTGCCAT	
980-2392F21	TAATTTCCTCATCGCTTTGCT	F-snp/R-snp primers for I71V in *Bcin16g00980*
980-3818R19	GTTTGCTCGATCACTCACC	
980-2716F20	GTATCTGGATCCGTTGCTTC	F-snp/R-snp primers for E395D in *Bcin16g00980*
980-3818R19	GTTTGCTCGATCACTCACC	
2880-1030F21	GGAAAGAAGCACATTTAGCCA	F-snp/R-snp primers for L412F in *Bcpos5*
2880-1555R21	AAACAGGAGATAGAATCAGCC	
820-ATG	ATGCGGCGCACCTTGA	Amplification primers for CDS of *Bcmdl1*
820-TAG	CTACTTCGTTCTGGTATTTGTC	
q820-1640F20	AATCTACTATCGGTTCGCTT	qRT-PCR primers for *Bcmdl1*
q820-1771R19	AGCCAGACATAAGAACAGG	
qTubA-F	TCTGCCATTTTGTAAGTTTGC	qRT-PCR primers for *BctubA*
qTubA-R	TTCTTGTTTTGGACGTTGC	
Hyg-HF	TCGACAGAAGATGATATTGAAGGAG	Amplification primers for whole hygromycin B resistance gene
Hyg-HR	GTTAAGTGGATCCGGCATCT	

HY-R	AGCATCAGCTCATCGAGAGCCT	Amplification primers for partial hygromycin B resistance gene
YG-F	AGGGCGAAGAATCTCGTGCTTT	
Hyg-GF	AGATCAGCCCACTTGTAAGCA	Check primers for whole hygromycin B resistance gene
Hyg-GR	TTCTACACAGCCATCGGTCCA	
Hyg-CF	AGGAATCGGTCAATACACTACAT	Check primers for insertion of hygromycin B resistance gene
Hyg-CR	ATGTAGTGTATTGACCGATTCCT	
Neo-F	GTCGACAGAAGATGATATTGAAGG	Amplification primers for whole neomycin resistance gene
Neo-R	TCTAGAAAGAAGGATTACCTCTAAAC	
NE-R	AAAAGCGGCCATTTTCCACCAT	Amplification primers for partial neomycin resistance gene
EO-F	GGGAAGGGACTGGCTGCTATTG	
Neo-GF	CCGGTCATACCTTCTTAAGTTCG	Check primers for whole neomycin resistance gene
Neo-GR	CACTCTTTGCTGCTTGGACA	
Neo-CF	TACCTGCCCATTCGACCAC	Check primers for insertion of neomycin resistance gene
Neo-CR	TGATCGACAAGACCGGCTTC	
820-763F21	AAATGCCAAAACAAGTAAGCC	F1/R1 primers for replacing/complementing *Bcmdl1*
820-6128R20-HFr	TCAATATCATCTTCTGTCGA-AGTTTAGATGACGAGCTCCA	
820-6187F21-HRr	AGATGCCGGATCCACTTAAC-GTTTCAATGTGGCATGAACCC	F2/R2 primers for replacing/complementing *Bcmdl1*
820-7690R20	GCCCTATTGTAATTGTTCTGCT	
820-1312F20	CTTGACATGAGTATGCACCC	F3/R3 primers for replacing/complementing *Bcmdl1*
820-7211R22	ATGCCCACTAACCATATTGACA	
820-5837F20	TACTCACAACTTCCGGCAAA	F4/R4 primers for replacing/complementing *Bcmdl1*
820-6433R19	TTGTCACCTCAAACGCAGA	
820-2527F20	TTACGACCTTTCAACAGTGC	F1/R1 primers for knocking out *Bcmdl1*
820-3417R20-HFr	TCAATATCATCTTCTGTCGA-GTTGATTGTATCTGCCAACC	
820-4357F20-HRr	AGATGCCGGATCCACTTAAC-CGTTTAAGTGATTCCGATGC	F2/R2 primers for knocking out *Bcmdl1*
820-6179R19	CACATTGAAACGGAACACC	
820-2589F20	TCACATCACACGAGAACGAC	F3/R3 primers for knocking out *Bcmdl1*
820-6128R20	AGTTTAGATGACGAGCTCCA	
820-1881F21	CTACAACAATTATCCTTTGCG	F4/R4 primers for knocking out *Bcmdl1*
820-5318R20	AGGTATCCAATTAGCACGTA	
820-186F20	CCTTCTTCGGTTGTAGCATC	F1/R1 primers for overexpressing *Bcmdl1*
820-765R20-HFr	TCAATATCATCTTCTGTCGA-TGGCTTACTTGTTTTGGCAT	
EF1-918F-HRr	AGATGCCGGATCCACTTAAC-TTCATCTTGTCTCGAGCTTG	F1ef/R1ef primers for overexpressing *Bcmdl1*
EF1-2388R22-820r	TCAAGGTGCGCCGCAT-TTTGATGGTTGTGTTTGTTTAT	
820-ATG	ATGCGGCGCACCTTGA	F2/R2 primers for overexpressing *Bcmdl1*
820-5188R20	GTTTTCCTTCCTCTCGGTCT	
820-221F21	AAGTTGTAACATACCGCCCTA	F3/R3 primers for overexpressing *Bcmdl1*
820-5140R19	CTCGAGCTTTCTTCGTCCA	

820-653F20	TTGAAGAACAAGCCAACACC	F4/R4 primers for overexpressing *Bcmdl1*
820-2297R20	CTTGAAAGCTACATCGCAGA	
z820-2219F21	ATCCAAGGATCCCCC-GTAGTACACAATCCGAGCATC	Amplification primers for Bcmdl1 to construct plasmid pCB-GFP-Bcmdl1
z820-4646R20	CTTGGAAACCATGAT-CTTCGTTCTGGTATTTGTCT	
GFP-ceF	TCTCTCTCTCTTCTTCTCTTCTTCCATCC	Check primers for plasmid pCB-GFP-Bcmdl1
GFP-ceR	CAATTTACCATATGTAGCGTCTCCCT	
820-6187F21-NRr	GTTTAGAGGTAATCCTTCTTTCTAGA-GTTTCAATGTGGCATGAACCC	F2/R2 primers for labeling Bcmdl1 with GFP
820-7690R20	GCCCTATTGTAATTGTTCTGCT	
820-ATG	ATGCGGCGCACCTTGA	F3/R3 primers for labeling Bcmdl1 with GFP
820-7211R22	ATGCCCACTAACCATATTGACA	
820-4573F20	CGTTTAAGTGATTCCGATGC	F4/R4 primers for labeling Bcmdl1 with GFP
820 6433R19	TTGTCACCTCAAACGCAGA	
820-1881F21	CTACAACAATTATCCTTTGCG	Checking primer for left flanking region of GFP transformants

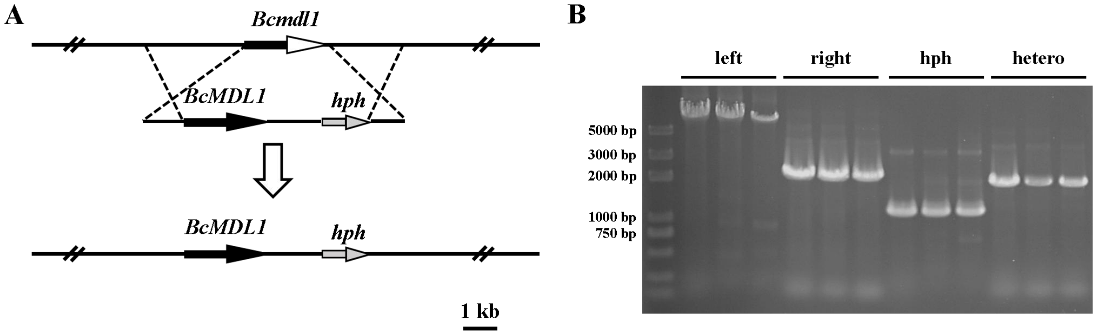

FIG S1 Construction and identification of E407K insertion transformants of *Bcmdl1* gene. (A) Mutation E407K insertion strategy of *Bcmdl1*. The hygromycin resistance cassette (hph), wild-type *Bcmdl1* (*Bcmdl1*), and *Bcmdl1* containing E407K mutation (*BcMDL1*) are denoted by the gray arrow, black and white arrow, and black arrow, respectively. (B) PCR analyses of insertion transformants of *Bcmdl1*. The transformants are *Bcmdl1*^{E407K}-1, 12, and 67, respectively. left: left region of hygromycin resistance cassette; right: right region of hygromycin resistance cassette; hph: hygromycin resistance cassette; hetero: heterozygote.

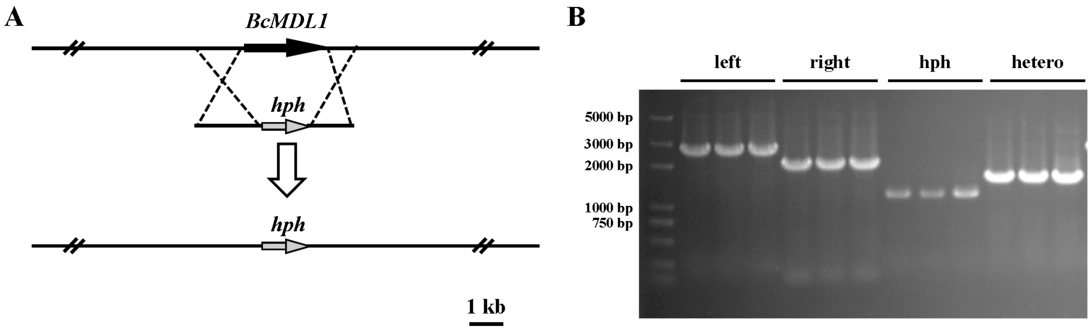

FIG S2 Construction and identification of knockout transformants of *Bcmdl1* gene. (A) Gene knockout strategy of *Bcmdl1*. The hygromycin resistance cassette (hph) and *Bcmdl1* containing E407K mutation (*BcMDL1*) are denoted by the gray arrow and black arrow. (B) PCR analyses of knockout transformants of *Bcmdl1*. The transformants are *ΔBcmdl1*-3, 13, and 37, respectively. left: left region of hygromycin resistance cassette; right: right region of hygromycin resistance cassette; hph: hygromycin resistance cassette; hetero: heterozygote.

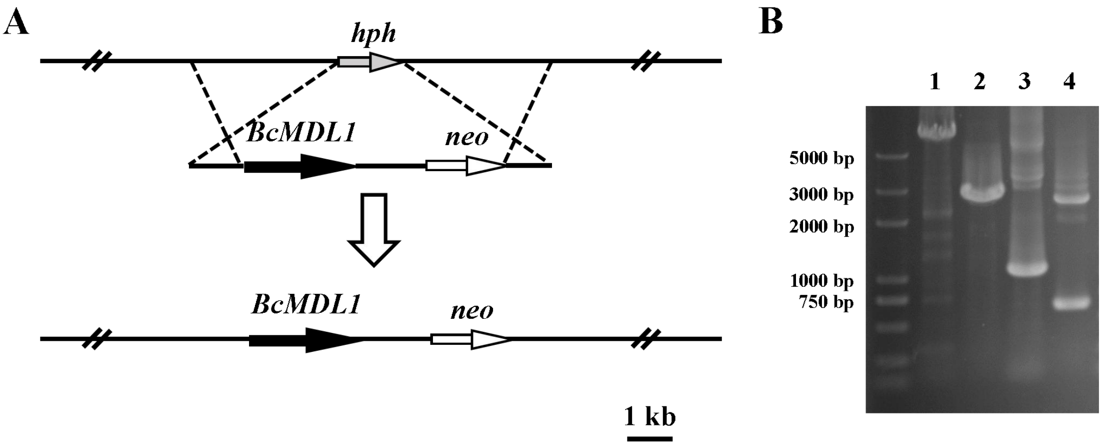

FIG S3 Construction and identification of complemented transformants of *Bcmdl1* gene. (A) Gene complement strategy of *Bcmdl1*. The hygromycin resistance cassette (hph), neomycin resistance cassette (neo), and *Bcmdl1* containing E407K mutation (*BcMDL1*) are denoted by the gray arrow, white arrow, and black arrow, respectively. (B) PCR analyses of complemented transformants of *Bcmdl1*. The transformant is *ΔBcmdl1*-37-C. 1: left region of neomycin resistance cassette; 2: right region of neomycin resistance cassette; 3: neomycin resistance cassette; 4: heterozygote.

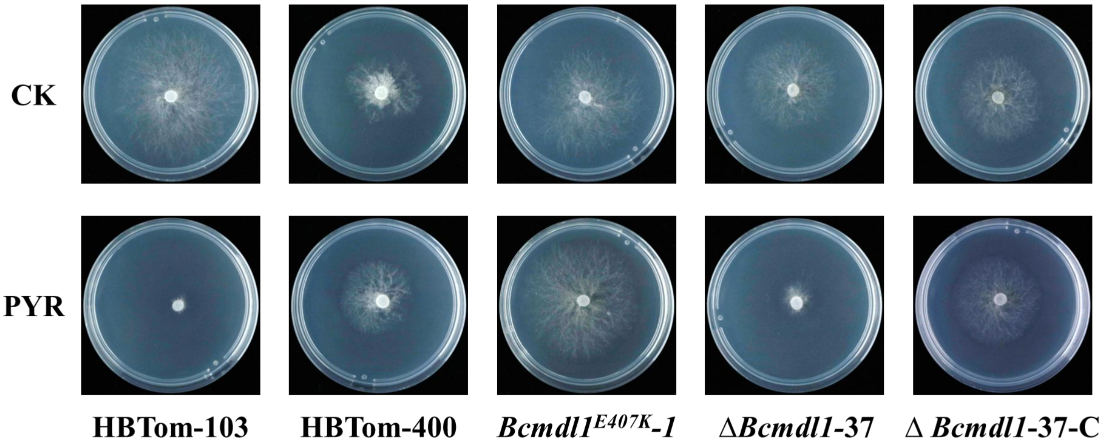

FIG S4 Sensitivity of E407K insertion, *Bcmdl1* knockout and complemented transformants to pyrimethanil. Isolates HBTom-400 and HBTom-103 were resistant and sensitive filed isolates to anilinopyrimidine fungicides. Transformant *Bcmdl1*^{E407K}-1 was E407K insertion transformant with HBTom-103 as parental isolate. Δ*Bcmdl1*-37 was knockout transformant of *Bcmdl1* with HBTom-400 as parental isolate. Transformant Δ*Bcmdl1*-37-C was complemented transformant of *Bcmdl1* using transformant Δ*Bcmdl1*-37 as the parental strain. CK and PYR were the fresh CzapeK-Dox agar and CzapeK-Dox agar amended with pyrimethanil at 10 μg/mL. The colonies were observed after three days of incubation at 20°C in darkness.

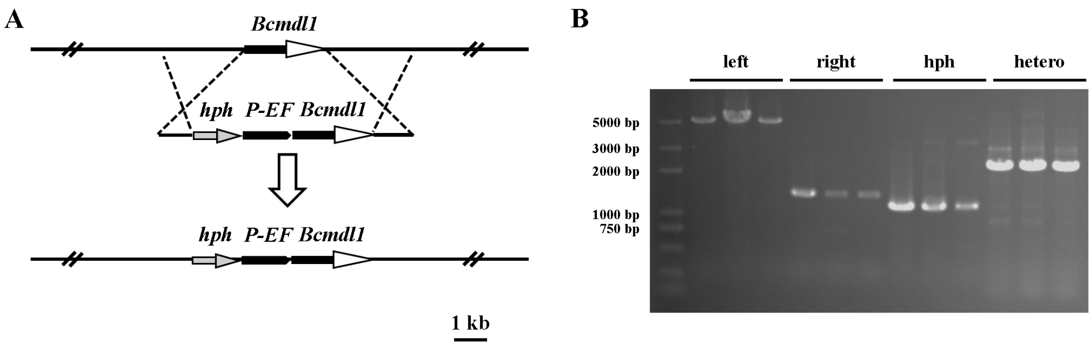

FIG S5 Construction and identification of overexpression transformants of *Bcmdl1* gene. (A) Gene overexpression strategy of *Bcmdl1*. The hygromycin resistance cassette (hph) and wild-type *Bcmdl1* (*Bcmdl1*) are denoted by the gray arrow and black and white arrow. (B) PCR analyses of overexpression transformants of *Bcmdl1*. The transformants are *ovBcmdl1*-3, 6, and 16, respectively. left: left region of hygromycin resistance cassette; right: right region of hygromycin resistance cassette; hph: hygromycin resistance cassette; hetero: heterozygote.

```
Botrytis cinerea      1   MRRTLTLGNGWGGASICDVAFKQVSKPIS-APAAARWTPSGPSLVAKRFSSGFKNSPSPL
Botryotinia calthae   1   MRRTLTLGNGWGAASICDIAFKQVSKPIP-APAAARWTPSGLTPVAKRFSSGFKNSPSPL
Fusarium oxysporum    1   ----MVSGVARRVLLPRLAEPSQVSLATSRCQLGASFHKSEALPLQSRLFHASSTRQAAL

Botrytis cinerea      60  SPASKPTPQLSTSRLLQQTRIGLS----SPWRPAVSQLRPFNSASPLQNSPKGSDRQDHI
Botryotinia calthae   60  SPASKPTPQLSTSRLLQQTRIGLS----SPWRPAVSQLRPFNSASPLQNSPKGSDRQDHI
Fusarium oxysporum    57  LPFSIATFRSSANNSLARNQPSLAHNRPSQFGPVSHALRQFSTAPRLLQEIQ-TKVEEGA

Botrytis cinerea      116 TRERQRREEEEEDNIDEQGDFARSEKASKATQVNLSARLQSSGKKQGNTAAFGEIWRLLK
Botryotinia calthae   116 TRERQRREEEEEDTIDEQGDFARSEKASKATQVNLSARLQSSGKKQGNTAAFGEIWRLLK
Fusarium oxysporum    116 KKPVELEANRAIAQDEHKQEFTKSEKAARAAQVNLAAKLSKEGKQAG-KAGTDEIVRLIK

Botrytis cinerea      176 IARPEAKWLGLAFVFLLISSSITMSIPFSIGKILDLATKDPSEGEKLFGLDISQFFIALA
Botryotinia calthae   176 IARPEAKWLGLAFVFLLISSSITMSIPFSIGKILDLATKDPSEGEKLFGLDISQFFIALA
Fusarium oxysporum    175 IARPEIRWLGIAFVFLVISSSVTMLIPFSVGRILDLATKGESEDVRLFGLTMNQFFFGLG

Botrytis cinerea      236 CVLTMGAAANYGRIIILRIVGERIVARLRSQLYRRTYVQNAEFFDANRVGDLISRLSSDT
Botryotinia calthae   236 CVLTMGAAANYGRIIILRIVGERIVARLRSQLYRRTYVQNAEFFDANRVGDLISRLSSDT
Fusarium oxysporum    235 TVLTIGAMANFGRVVLLRIVGERVVARLRSQLYRRTYVQDAEFFDANRVGDLISRLSSDT

Botrytis cinerea      296 VIVGKSITQNLSDGLRAIVSGGAGFTAMAWVSLKLTSILCLMFPPVAIGAFFYGRAIRNL
Botryotinia calthae   296 VIVGKSITQNLSDGLRAIVSGGAGFTAMAWVSLKLTSILCLMFPPVAIGAFFYGRAIRNL
Fusarium oxysporum    295 VIVGKSVTQNLSDGLRALFSGGAGFAIMVWTSPKLTGLLLLMFPPIAVGAFIYGRAIRNV

Botrytis cinerea      356 SRKIQRNLGTLTKIAEERLGNVRTSQAFAGETQEVGRYNQQIKKIFSLGKREALISATFF
Botryotinia calthae   356 SRKIQRNLGTLTKIAEERLGNVRTSQAFAGETQEVGRYNQQIKKIFSLGKREALISATFF
Fusarium oxysporum    355 SRSIQKNLGTLTKIAEERLGNVKTSQAFVGEVQEIGRYNNQIRKIFALGKKESLIAATFF

Botrytis cinerea      416 SSSGFFGNMTILALLYTGGSMVKNGMISIGELTSFLMYTAYAGSSLFGVSSFYSELMKGV
Botryotinia calthae   416 SSSGFFGNMTILALLYTGGSMVKNGMISIGELTSFLMYTAYAGSSLFGVSSFYSELMKGV
Fusarium oxysporum    415 ASTGWAGNMTILAMLIVGGGFVRSGAMSLGDLTSFMMYTAFAGSSLFGLSGFYSELMKGV

Botrytis cinerea      476 GAASRLFELQDRKPTIPATVGTKVKSAQGVIKFHNVSFAYPTRPAVTIFDGLDFEIPSGT
Botryotinia calthae   476 GAASRLFELQDRKPTIPATVGTKVKSAQGVIKFHNVSFAYPTRPAVTIFDGLDFEIPSGT
Fusarium oxysporum    475 GAASRLFELQDRKPGIPQTVGVRVKSAQGPIKFTDVSFAYPTRPAVKIFNGLDFEIPSGS

Botrytis cinerea      536 NVAIVGPSGGGKSTIGSLLLRFYNPTEGKITINGQDITKMNGKSLRRRIGMVGQEPVLMS
Botryotinia calthae   536 NVAIVGPSGGGKSTIGSLLLRFYNPTEGKITINGQDITKMNGKSLRRRIGMVGQEPVLMS
Fusarium oxysporum    535 NVCVVGPSGGGKSTVASLLLRFYNPTSGTITINGQDISGMNVKSLRRRIGMVSQEPVLFS

Botrytis cinerea      596 GSVAENIAYGKPHASRSEIIAAARKANCQFIGDFPEGLDTQVGARGAQLSGGQKQRIAIA
Botryotinia calthae   596 GSVAENIAYGKPHASRSEIIAAARKANCQFIGDFPEGLDTQVGARGAQLSGGQKQRIAIA
Fusarium oxysporum    595 GTIAENIAYGKPQASRFDIISAARRANCNFISDLPDGLETQVGARGSQLSGGQKQRIAIA

Botrytis cinerea      656 RALLKNPDILILDEATSALDAESETLVNSALAALLRGHNTTISIAHRLSTIKRSDHIIVL
Botryotinia calthae   656 RALLKNPDILILDEATSALDAESETLVNSALAALLRGHNTTISIAHRLSTIKRSDHIIVL
Fusarium oxysporum    655 RALLKDPDILILDEATSALDAESETLVNEALAGLLRGRNTTISIAHRLSTIKRSDQIIVL

Botrytis cinerea      716 GNDGKVAETGTYNSLSNNPNSAFSKLMEWQMSGGDAA-DHRPVELEGHFTESEEISDDLS
Botryotinia calthae   716 GNDGKVAETGTYNTLSNNPNSAFSKLMEWQMSGGDAA-DHRPVELEGHFTESEEISDDLS
Fusarium oxysporum    715 NSEGKVAEIGSYKQLASDKDSAFSKLMEWQMSGGEVSKDGGSTASRAHITEAEELEDDLE

Botrytis cinerea      775 RLSDSDATEGEAIEE--------KTKTEAVLEKTNTRTK
Botryotinia calthae   775 RLSDSDATEGETIEEGTRERKENKTKTEAVLEKTNTRTK
Fusarium oxysporum    775 RDDEEENIEHEEESR--------DSKNEEKRP-------
```

FIG S6 Amino acid sequences of Bcmdl1 aligned with Mdl1 proteins from *Botryotinia calthae* (TEY42091.1) and *Fusarium oxysporum* (RKL18179.1).

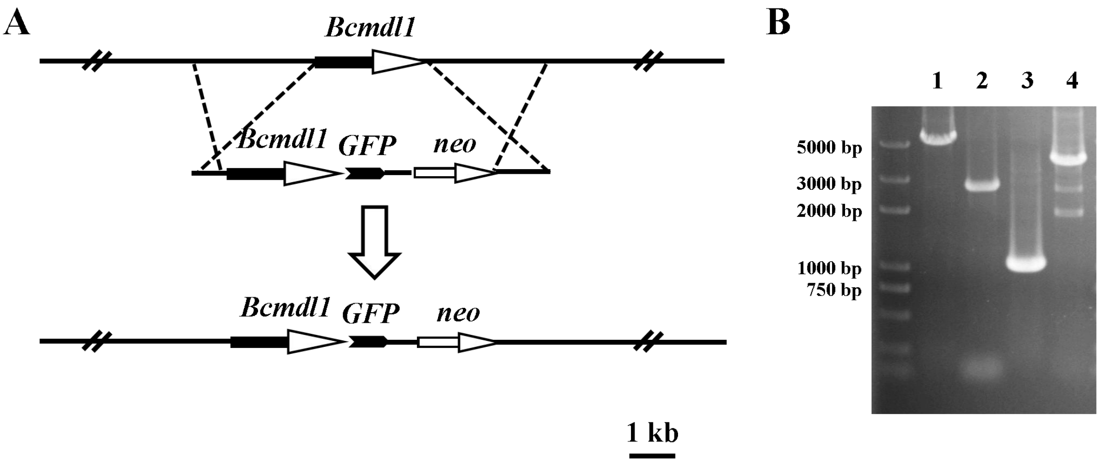

FIG S7 Construction and identification of green fluorescent protein (GFP) labeled transformants of *Bcmdl1* gene. (A) Gene GFP-labelling strategy of *Bcmdl1*. The neomycin resistance cassette (neo), wild-type *Bcmdl1* (*Bcmdl1*), and GFP are denoted by the white arrow, black and white arrow, and polygon, respectively. (B) PCR analyses of GFP labeled transformant of *Bcmdl1*. The transformant is Bcmdl1::GFP-3. 1: left region of neomycin resistance cassette; 2: right region of neomycin resistance cassette; 3: neomycin resistance cassette; 4: heterozygote.

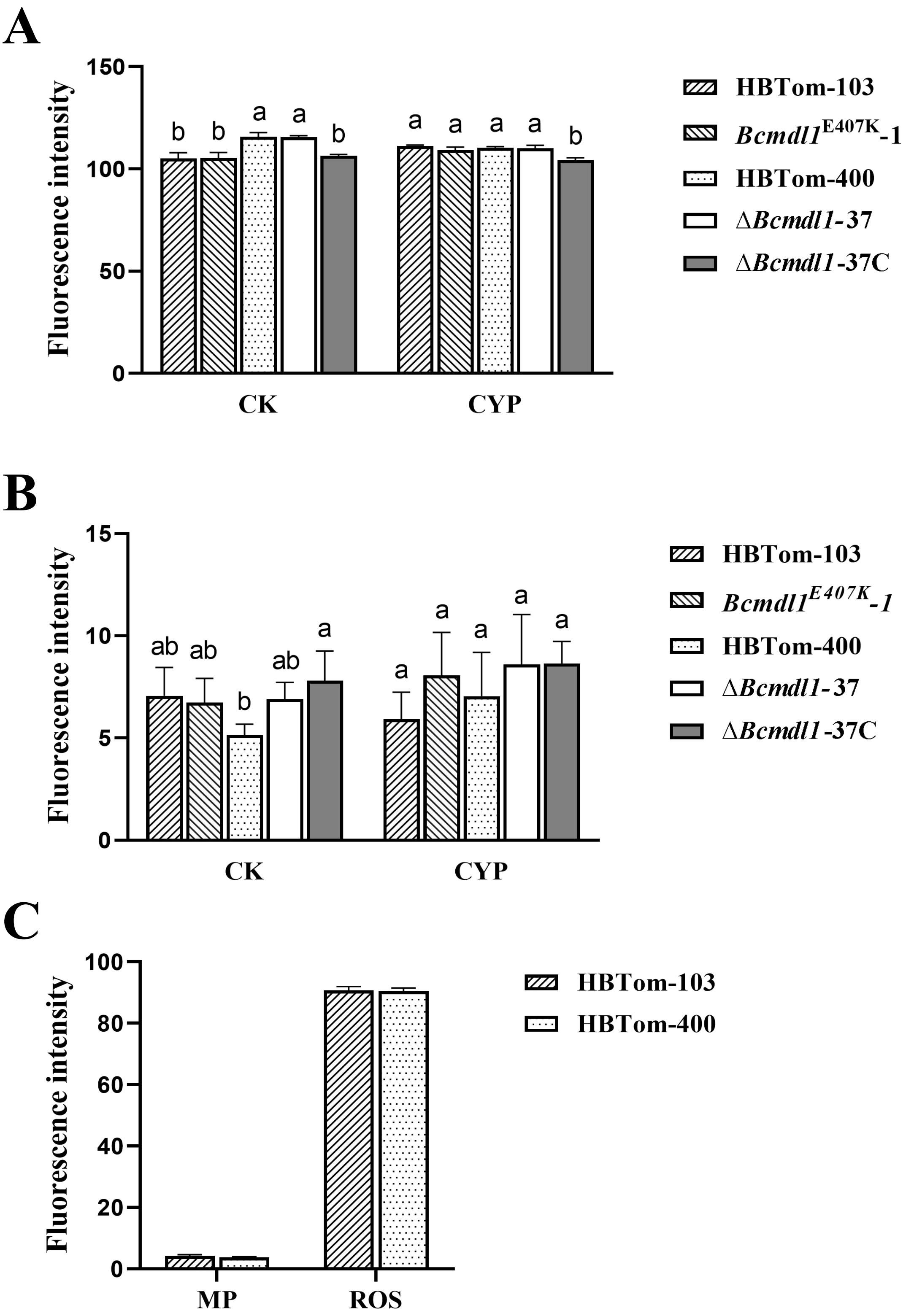

FIG S8 Bcmdl1 is not related to reactive oxygen species (ROS) and mitochondrial membrane potential (MP). (A) Contents of ROS in knockout and E407K insertion transformants of Bcmdl1 with or without cyprodinil treatment. (B) Contents of MP in knockout and E407K insertion transformants of Bcmdl1 with or without cyprodinil treatment. (C) Positive control of MP and ROS using parental isolates. Cell permeant reagent 2'-7'dichlorofluorescin diacetate (DCFH-DA) was used as positive control of ROS at 10 mM for two hours. Carbonyl cyanide m chlorophenyl hydrazone (CCCP)

was used as positive control of MP at 10 mM for four hours. HBTom-103 and HBTom-400 were the parental isolates of E407K insertion and knockout transformants of Bcmdl1, respectively. Transformants Bcmdl1E407K-1, ΔBcmdl1-37, and ΔBcmdl1-37C were E407K insertion, knockout and complementary transformants of Bcmdl1, respectively. CK was the control without cyprodinil treatment whereas CYP was the treatment with cyprodinil. Fluorescence intensity is the mean grey-scale values in images processed by LAS X. Different letters indicated significant differences ($P<0.05$) according to one-way analysis of variance (ANOVA) using the least significant difference (LSD). Means and standard errors were calculated from three repeats.
